# Monocyte-derived inflammatory Langerhans cells and dermal dendritic cells mediate psoriasis-like inflammation

**DOI:** 10.1038/ncomms13581

**Published:** 2016-12-16

**Authors:** Tej Pratap Singh, Howard H. Zhang, Izabela Borek, Peter Wolf, Michael N. Hedrick, Satya P. Singh, Brian L. Kelsall, Bjorn E. Clausen, Joshua M. Farber

**Affiliations:** 1 Laboratory of Molecular Immunology, National Institute of Allergy and Infectious Diseases, US National Institutes of Health, Bethesda, Maryland 20892, USA; 2 Institute of Pathophysiology and Immunology, Medical University of Graz, 8010 Graz, Austria; 3 Department of Dermatology, Medical University of Graz, A-8036 Graz, Austria; 4 Institute for Molecular Medicine, University Medical Center of the Johannes Gutenberg-University Mainz, 55131 Mainz, Germany

## Abstract

Dendritic cells (DCs) have been implicated in the pathogenesis of psoriasis but the roles for specific DC subsets are not well defined. Here we show that DCs are required for psoriasis-like changes in mouse skin induced by the local injection of IL-23. However, Flt3L-dependent DCs and resident Langerhans cells are dispensable for the inflammation. In epidermis and dermis, the critical DCs are TNF-producing and IL-1β-producing monocyte-derived DCs, including a population of inflammatory Langerhans cells. Depleting Ly6C^hi^ blood monocytes reduces DC accumulation and the skin changes induced either by injecting IL-23 or by application of the TLR7 agonist imiquimod. Moreover, we find that IL-23-induced inflammation requires expression of CCR6 by DCs or their precursors, and that CCR6 mediates monocyte trafficking into inflamed skin. Collectively, our results imply that monocyte-derived cells are critical contributors to psoriasis through production of inflammatory cytokines that augment the activation of skin T cells.

## Abstract (web summary)

Imiquimod exacerbates IL-23-induced skin inflammation and models psoriasis in mice. Here the authors show that this pathology is not dependent on resident dendritic cells, but on CCR6-induced immigration of monocyte-derived cells.

Dendritic cells (DCs) are critical contributors to immune responses by bridging innate and adaptive immunity, and have been investigated for their roles in skin immunity. Plasmacytoid DCs (pDCs), conventional DCs (cDCs), Langerhans cells (LCs) and inflammatory DCs (iDCs), can be identified based on surface phenotype and developmental origin^1^,^2^. DC populations in the skin have been best characterized in mice^1^,^2^. Two major subsets of cDCs have been characterized in the mouse dermis, CD103^+^CD11b^−^ (CD103^+^) and CD103^−^CD11b^+^ (CD11b^+^) cells, whereas the epidermis contains Langerhans cells (LCs)^1^,^2^. Dermal CD103^+^ cDCs and CD11b^+^ cDCs are developmentally related to the lymphoid CD8α^+^ and CD8α^−^ cDCs, respectively, which reside in secondary lymphoid tissues together with tissue migratory DCs (tDCs)^1^,^2^. The functional homologues of mouse CD103^+^ and CD11b^+^ cDCs in humans are CD141^+^ and CD1c^+^ cDCs, respectively^2^.

The development and differentiation of mononuclear phagocytes require distinct signals. For example, Fms-related tyrosine kinase ligand 3 (Flt3L) is indispensable for cDCs, but is not required for the development of monocyte-derived DCs (moDCs), macrophages and LCs^1^,^2^. Also, the basic leucine zipper transcription factor ATF-like 3 (Batf3) is expressed by all cDCs but is selectively required for the generation of CD103^+^ CD11b^−^ cDCs^1^,^2^. The iDCs are found at inflammatory sites and arise from blood monocytes or other progenitors in both mice and humans^1^,^2^.

Monocytes form a heterogeneous population of cells that circulate between blood, spleen and bone marrow under steady-state conditions. Mouse monocytes can be subdivided into Ly6C^hi^ classical and Ly6C^low^ non-classical monocytes (corresponding to CD14^hi^ and CD14^low^ cells in humans)^2^,^3^. Monocytes are rapidly recruited to inflammatory sites and give rise to a variety of cell populations. In the skin, these populations include dermal macrophages, dermal DCs (monocyte-derived dermal DCs, moDDCs) and epidermal LCs (monocyte-derived LCs, moLCs)^1^,^4^,^5^. In monocyte trafficking, CCR2 has a critical role in the egress of monocytes from the bone marrow, but CCR2 is often not essential for the migration of monocytes to peripheral inflammatory sites^6^.

Among the inflammatory disorders of the skin, psoriasis is remarkable as a common, chronic autoimmune/autoinflammatory disease that bears similarities in its underlying mechanisms to other autoimmune diseases^7^. Psoriasis is characterized by increased proliferation and abnormal differentiation of keratinocytes, thickening of the epidermis, formation of new blood vessels and accumulation of leukocytes in epidermis and dermis, of which T cells and dendritic cells are the most critical^7^,^8^. Psoriatic skin contains large numbers of iDCs that are potent T-cell activators^7^,^9^. However, despite strong evidence implicating DCs in psoriasis, the contribution of specific DC subsets to disease pathogenesis remains undefined.

Both in psoriasis and in relevant mouse models, the IL-23/IL-17/IL-22 axis has a major role in disease^7^,^10^,^11^,^12^. IL-23, a cytokine required for the expansion and survival of pathogenic Th17 cells, is highly expressed in psoriatic skin and in mouse models, including a model that depends on the topical application of the TLR7 agonist, imiquimod (IMQ), in which IL-23 is produced by DCs^7^,^10^,^13^,^14^. Moreover, polymorphisms in the genes encoding the IL-23 receptor and the p19 and p40 subunits of IL-23 are associated with psoriasis susceptibility^7^, and blocking IL-23 is effective in treating established disease^15^. Both intradermal injection of IL-23 and the topical application of IMQ induce psoriasis-like keratinocyte proliferation, thickening of epidermis and dermal inflammation^7^,^15^,^16^,^17^,^18^, which is mediated by IL-22 and IL-17A^7^,^11^,^16^,^18^. IL-23, along with IL-12 and TNF produced by DCs, activates αβ and γδ T cells to produce IL-22 and IL-17A/F, which in turn stimulate keratinocyte proliferation, and release of S100-proteins, β-defensins, growth factors and chemokines that further contribute to disease^7^. In a recent comparison of skin transcriptomes in several mouse models of psoriasis, the transcriptome in the IL-23 injection model was the one that most closely matched the pattern of gene expression found in psoriatic skin^19^. Among inflammatory mediators, IL-1 family cytokines, including IL-1β, have also been implicated in psoriasis and in relevant mouse models^20^,^21^,^22^.

The recruitment of leukocytes to the skin is a critical component of psoriasis, and published data suggest that the chemokine receptor CCR6 is an important contributor to this process. CCR6 is expressed on all T cells that produce IL-17A/F and IL-22; has been implicated in the trafficking of cDCs, pDCs, LCs and monocytes into and/or within the skin; and is increased in psoriatic lesions—as is the CCR6 ligand, CCL20 (refs 3, 5, 12, 23, 24, 25, 26, 27, 28, 29, 30). In addition, we have shown previously that CCR6 is required for the psoriasis-like skin changes after injection of IL-23 (refs 17, 24, 31). Here we show that psoriasis-like pathology induced either by direct injection of IL-23 or by the application of IMQ requires inflammatory moDCs, and that, at least in the IL-23 injection model, CCR6 is important for the accumulation of these cells through its role in monocyte recruitment.

## Results

### Identification of DC subsets in inflamed skin

**Figure 1:**
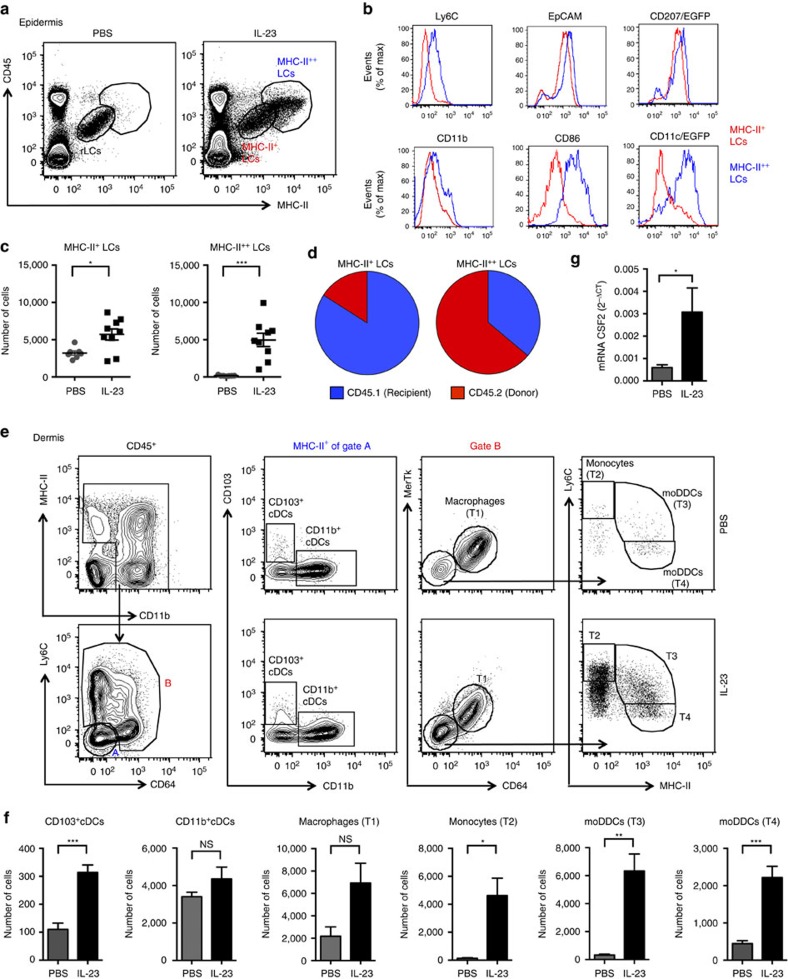
DC subsets accumulate in the skin after IL-23 injections. (**a**) Flow cytometry plots of cells prepared from epidermis of ears on day 6 after intradermal injections of PBS (left) or IL-23 (right) on days 1, 3 and 5 and stained for CD45 and MHC-II for identifying LCs, which are separated into regions based on expression of MHC-II. These are pooled samples from two ears. (**b**) Flow cytometry histograms of MHC-II^+^ and MHC-II^++^ LCs on day 6 from IL-23-injected ears, showing staining for Ly6C, EpCAM, CD11b and CD86, and expression of CD207/EGFP and CD11c/EGFP. (**c**) MHC-II^+^ and MHC-II^++^ LCs per ear on day 6 from PBS- and IL-23-injected ears. Each symbol represents a sample with cells from two to three ears. (**d**) Pie charts showing contributions of donor-derived and recipient-derived cells to the populations of MHC-II^+^ and MHC-II^++^ LCs in the ears of irradiated CD45.1 mice transplanted with CD45.2 bone marrow on day 6 following injections with IL-23. (**e**) Flow cytometry plots, and gating strategy for the identification of dermal cDCs (CD103^+^cDCs and CD11b^+^cDCs), macrophages (T1), monocytes (T2) and moDDCs (T3 and T4) on day 6 from PBS- and IL-23-injected ears. The data shown are from individual ears, which is true for all flow cytometry plots in the remainder of the manuscript. (**f**) Dermal macrophages, monocytes and DCs per ear on day 6 from PBS- and IL-23-injected ears. Samples that were analysed contained cells from one to four ears. (**g**) Expression of mRNA encoding CSF2 versus *Gapdh* mRNA on day 6 from IL-23-injected ears. Data are from one experiment representative of eight to ten experiments (**a**,**b**,**e**); or four experiments with a total of 16 PBS and 24 IL-23 mice (**c**); or two experiments with a total of four mice (**d**); or three experiments with a total of 10 PBS and 20 IL-23 mice (**f**); or two experiments with a total of six mice per group (**g**). All the data shown in this and the figures below came from samples from individual mice unless stated otherwise. The data are presented as mean±s.e.m. *P<0.05, **P<0.01, ***P<0.001 (unpaired Student's *t*-test).

To understand the roles for DCs in the psoriasis-like skin inflammation, we initially characterized the mononuclear phagocytes that accumulated after 6 days of every-other-day intradermal injections of IL-23. The Langerhans cells were identified within the epidermis based on expression of CD45, MHC-II and CD207/langerin, and were characterized further based on the expression of additional markers^1^. Epidermis from PBS-injected ears harboured only one major population of Langerhans cells, which we designated as resident (r)LCs. However, IL-23-injected ears contained a more complex population of LCs that could be separated into two subsets based on expression of MHC-II, which we designated MHC-II^+^ and MHC-II^++^ LCs (Fig. 1a). Although the MHC-II^+^ gate includes rLCs, in the inflamed ear not all MHC-II^+^ LCs are rLCs (see below). The majority of MHC-II^++^ LCs expresses high levels of CD11c and CD86, increased levels of Ly6C, and similar levels of CD11b, EpCAM and CD207/langerin as compared with MHC-II^+^ LCs (Fig. 1b). Injection of IL-23 resulted in a small increase in the number of MHC-II^+^ LCs and a large increase in MHC-II^++^ LCs (Fig. 1c).

Bone marrow chimera experiments revealed that following IL-23 injections, the MHC-II^+^ LCs, which contained the radioresistant rLCs from the recipient mice, also contained a smaller number of donor-derived cells. Approximately 30–40% of the MHC-II^++^ LCs were derived from radioresistant/recipient cells, possibly rLCs, with the remaining cells derived from donor bone marrow (Fig. 1d; Supplementary Fig. 1a–c,e). Consistent with their originating from monocytes, the donor-derived MHC-II^+^ and MHC-II^++^ LCs showed expression of Ly6C, and depletion of Ly6C^hi^ monocytes almost eliminated the recruitment of donor-derived, but not recipient-derived LCs (Supplementary Fig. 1c–e). Recent evidence suggests that under inflammatory conditions, monocytes recruited to the skin can proliferate and differentiate into LCs^4^,^5^.

Next, to identify DCs and other mononuclear phagocytes in the dermis, we analysed dermal CD45^+^ cells that expressed MHC-II and/or CD11b. The cDCs were then identified as cells expressing CD103 or CD11b within the MHC-II^+^Ly6C^−^CD64^−^ population (Fig. 1e). We used the combination of Mer tyrosine kinase (MerTK) and CD64 to identify macrophages (T1) among the remaining cells that stained for Ly6C and/or CD64 (Fig. 1e, Gate B). MerTK selectively identifies macrophages, and CD64 (FcγR1) is an additional macrophage marker^32^,^33^. Monocytes and moDDCs were separated among the remaining MerTK^−^CD64^−^ cells based on their relative expression of Ly6C and MHC-II. We identified monocytes as Ly6C^hi^MHC-II^−^ (T2), and moDDCs as Ly6C^int^MHC-II^hi^ (T3) and Ly6C^low^MHC-II^hi^ (T4; ref. 33; Fig. 1e; cells that were Ly6C^int-low^MHC-II^−^ are neutrophils). Numbers of CD103^+^ cDCs, monocytes and moDCs in the dermis were significantly increased after injections of IL-23, with the largest absolute increases in numbers of monocytes and moDDCs (Fig. 1e,f). Bone marrow chimera experiments demonstrated that all dermal myeloid cells in the IL-23-injected ears were derived from radiosensitive bone marrow precursors, and depleting Ly6^hi^ monocytes eliminated the monocytes and moDDCs (Supplementary Fig. 1f,g). Notably, the messenger RNA (mRNA) for GM-CSF (CSF2), a cytokine that is used routinely for generating DCs from monocytes *in vitro*^1^, was increased in the skin after IL-23 injection, possibly contributing to the differentiation of monocytes into moDCs (Fig. 1g).

Intradermal injection of IL-23 also increased the accumulation of MHC-II^hi^CD11c^+^ tDCs in the skin-draining lymph node (Supplementary Fig. 2a). To address the importance of tDC migration into the lymph node we used *Ccr7*^*−/−*^ mice, in which tDCs are unable to migrate^1^. Wild-type (WT) and *Ccr7*^*−/−*^ mice showed similar levels of skin inflammation in the IL-23 injected ears (Supplementary Fig. 2b,c), suggesting that homing of tDCs into the skin-draining lymph nodes is not critical for the IL-23-induced skin inflammation.

### Depletion of DCs prevents skin inflammation

**Figure 2:**
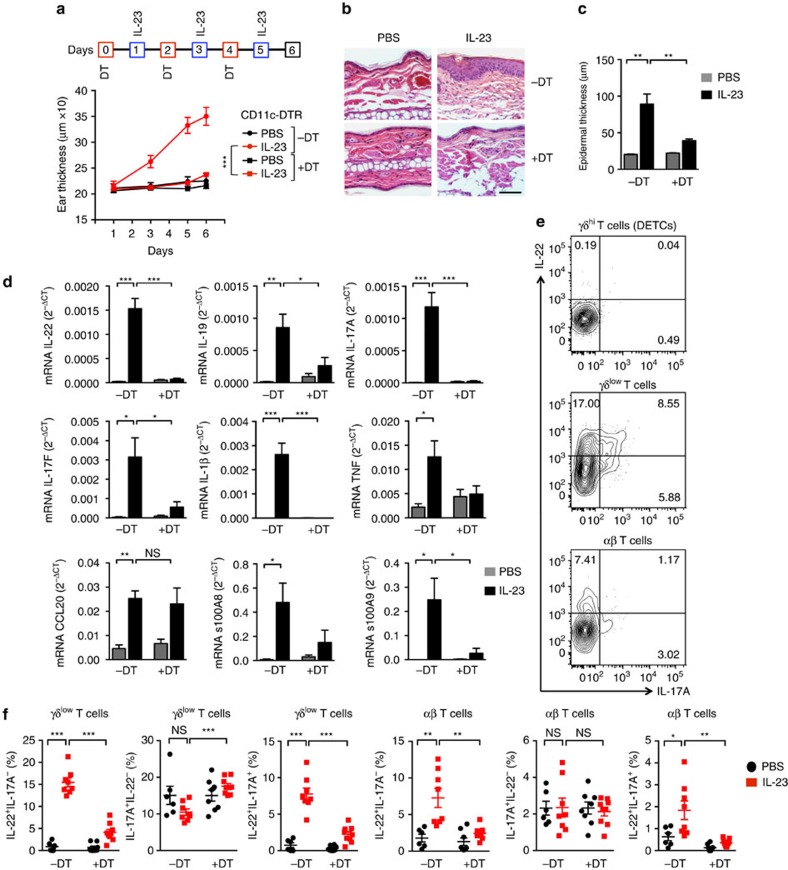
Depletion of CD11c-expressing cells protects from IL-23-induced changes. (**a**) Ear thickness for CD11c-DTR mice treated without (−DT) or with (+DT) diphtheria toxin and injected in the ears with IL-23 as shown. PBS was injected into the ears of appropriate control mice at the same times as IL-23 (not indicated). (**b**) Skin histology on day 6. Scale bar, 200 μm. (**c**) Epidermal thickness of ear skin on day 6. (**d**) Expression of ear mRNAs encoding proteins as indicated versus *Gapdh* mRNA on day 6. (**e**) Flow cytometry plots of cells prepared from IL-23-injected ears on day 6 following treatment of the cells *ex vivo* with leukocyte-activating cocktail for 4 h, surface staining for CD45, CD3ɛ, γδTCR and βTCR, and intracellular staining for IL-17A and IL-22. Only CD45^+^ CD3ɛ^+^ cells are shown. Numbers indicate percentages of cells within the quadrants, and quadrants are based on staining with isotype-matched control antibodies. (**f**) Frequencies of the T cells expressing IL-22 and/or IL-17A from day 6 ears. The cells were activated and stained as in **e**. The data are displayed from one experiment representative of four with a minimum of 16 mice total in each group (**a**; mean±s.d.; pairwise statistical comparison was done between IL-23-injected mice±DT on day 6, using mice from all the experiments); or one experiment representative of two (**b**); or two experiments with a total of five PBS (±DT) and six IL-23 (±DT) mice (**c**); or two experiments with a total of six mice per group (**d**); or one experiment representative of five (**e**); or three experiments with a minimum of six mice total per group (**f**). The data are presented as mean±s.e.m. unless otherwise noted. NS, not significant; *P<0.05, **P<0.01, ***P<0.001 (unpaired Student's *t*-test).

To test the functional role for DCs in the IL-23-induced skin changes, we depleted the CD11c-expressing cells by injecting diphtheria toxin (DT) into mice expressing a diphtheria toxin receptor (DTR)-enhanced green fluorescent protein (EGFP) fusion protein under control of the *Itgax* (CD11c) promoter (CD11c-DTR; Fig. 2a). Using the DTR/EGFP reporter, we detected *Itgax* expression, at various levels, specifically in DCs - in CD103^+^cDCs, CD11b^+^cDCs, moDDCs, MHC-II^+^LCs and MHC-II^++^LCs—with little detected in macrophages or monocytes (Fig. 1b and Supplementary Fig. 3a). Accordingly, the LCs (MHC-II^+^ LCs and MHC-II^++^ LCs), cDCs and moDDCs—but not macrophages or monocytes—were depleted after every-other-day administration of DT (Supplementary Fig. 3b,c). Remarkably, DT-treated CD11c-DTR mice were protected from IL-23-induced skin changes (Fig. 2). The mice depleted of CD11c-expressing cells showed no ear swelling (Fig. 2a) or microscopic pathology such as epidermal thickening (Fig. 2b,c). The depleted mice also showed no increases in mRNAs encoding IL-10 family members such as IL-22 and IL-19, the IL-17 family members IL-17A and IL-17F, IL-1β, TNF and the calprotectin subunits s100A8 and s100A9, although the increase in CCL20 mRNA was not affected (Fig. 2d). All these genes/proteins have been implicated in the pathogenesis of psoriasis and can be induced after injection of IL-23 in mouse skin^7^,^16^,^17^,^19^,^30^.

### Dermal γδ T cells are the initial responders to IL-23

Both myeloid cells, including DCs, as well as lymphocytes, have been reported to express the IL-23 receptor in lymph nodes and/or peripheral tissues^34^. To identify cells that might be responding directly to injected IL-23, we investigated *Il23r* expression on cells in the skin using reporter mice^34^. We found that *Il23r* was limited to the γδ^low^ T cells and αβ T cells (Supplementary Fig. 4a). Moreover, within 18 h after injection of IL-23, the γδ^low^ (but not the αβ) T cells were making IL-17A *in vivo*, as detected using *Il17a* reporter mice, suggesting that these cells are the first to respond (Supplementary Fig. 4b).

Although IL-17A has a role in the IL-23 induced dermatitis, IL-22 is a primary driver of the pathology^11^,^16^. However, at 18 h after one injection of IL-23, we could not detect T cells capable of making IL-22 (Supplementary Fig. 4c). In analysing cells from ears on Day 6, we found that both γδ^low^ and αβ T cells but not DETC (γδ^hi^ T cells), were able to produce IL-17A and IL-22 after activation *ex vivo* (Fig. 2e). Unlike IL-17A, which could be made by some T cells from PBS-injected ears after activation *ex vivo*, IL-22 could only be made by T cells isolated from ears injected with IL-23. In addition, DT treatment of IL-23-injected CD11c-DTR mice significantly reduced the numbers of cells able to make IL-22, suggesting that acquiring this critical capability required DCs (Fig. 2f, Supplementary Fig. 5).

### Flt3L-dependent DCs are dispensable for inflammation

**Figure 3:**
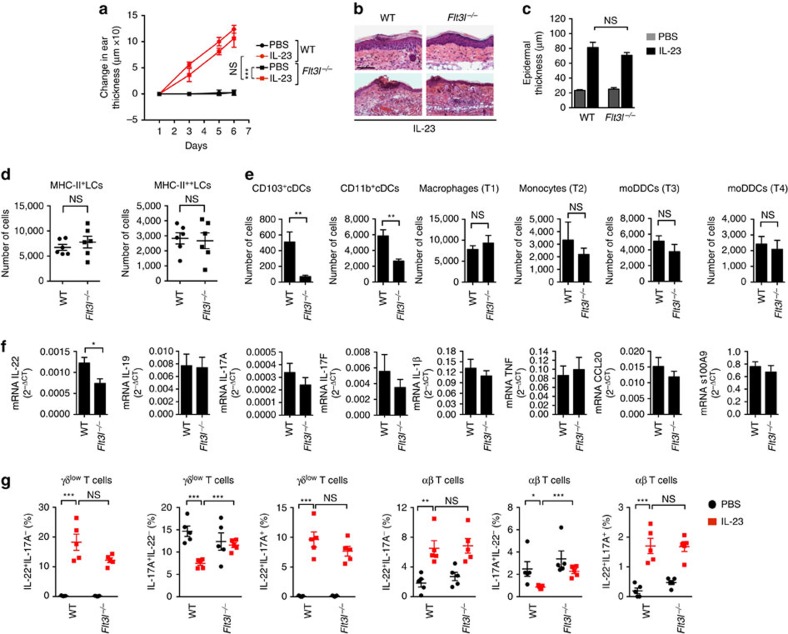
Flt3L-dependent DCs are not required for IL-23-induced inflammation. (**a**) Changes in the ear thickness for WT and *Flt3l*^*−/−*^ mice injected in the ears with PBS or IL-23 on days 1, 3 and 5. (**b**) Skin histology on day 6 at two magnifications. Scale bar, 200 μm. (**c**) Epidermal thickness of ear skin on day 6. (**d**) MHC-II^+^ and MHC-II^++^ LCs per ear on day 6 from IL-23-injected ears. Each symbol represents a sample with cells from one to three ears. (**e**) Dermal macrophages, monocytes and DCs per ear, and (**f**) expression of ear mRNAs encoding proteins as indicated versus *Gapdh* mRNA on day 6 in IL-23-injected ears. The samples that were analysed in **e** contained cells from one or two ears. (**g**) Frequencies of the T cells expressing IL-22 and/or IL-17A on day 6. The cells were activated and stained as in Fig. 2e. The data are displayed from one experiment representative of two with a total of nine mice per group (**a**; mean±s.d.; pairwise statistical comparisons were done at day 6 using mice from all the experiments); or one experiment representative of three (**b**); or three experiments with a total of 11 WT and 15 *Flt3*^*−/−*^ mice (**c**); or three experiments with a total of 10 WT and nine *Flt3*^*−/−*^ mice (**d**); or three experiments with a total of 10 WT and seven *Flt3*^*−/−*^ mice (**e**); or two experiments with a total of seven mice per group (**f**); or one experiment representative of three with a total of nine PBS and 11 IL-23 mice per genotype (**g**). The data are presented as mean±s.e.m. unless otherwise noted. NS, not significant; *P<0.05, **P<0.01, ***P<0.001 (unpaired Student's *t*-test).

Having demonstrated that DT-treated CD11c-DTR mice were protected from IL-23-induced changes, we next investigated which type(s) of cutaneous DCs were most important in supporting the inflammation. To determine whether or not cDCs were dispensable, we used *Flt3l*^*−/−*^ mice, which have much diminished numbers of cDCs and pDCs^1^. IL-23 injections in the *Flt3l*^*−/−*^ mice resulted in slightly less ear swelling and epidermal thickening as compared with the WT mice, although these differences were not significant (Fig. 3a–c). The analysis of cells from the IL-23-injected ears of *Flt3l*^*−/−*^ mice showed similar numbers of MHC-II^+^ LCs and MHC-II^++^ LCs in the epidermis, and monocytes, moDDCs and macrophages in the dermis as compared with WT mice, but, as expected, significantly fewer cDCs, both in the CD103^+^ and CD11b^+^ subsets (Fig. 3d,e). IL-23-injected ears from the *Flt3l*^*−/−*^ mice showed a decrease in the mRNAs for IL-22 as compared with WT mice, but not of the other relevant mRNA (Fig. 3f). In addition, numbers of T cells in the IL-23-injected skin that were able to produce IL-22 and IL-17A after activation *ex vivo* were not reduced significantly in *Flt3l*^*−/−*^ as compared with WT mice, although there was a suggestion of a decrease in the number of IL-22^+^IL-17A^−^ and IL-22^+^IL-17A^+^ γδ^low^ cells, associated with a redistribution into, and increase in the γδ^low^ IL-17A^+^IL-22^−^ subset (Fig. 3g). Together, these results demonstrated that Flt3L-dependent DCs were not the major DC contributors to the IL-23-induced skin pathology, and led us to investigate the roles for Flt3L-independent DCs.

### moDCs are the principal contributors to inflammation

**Figure 4:**
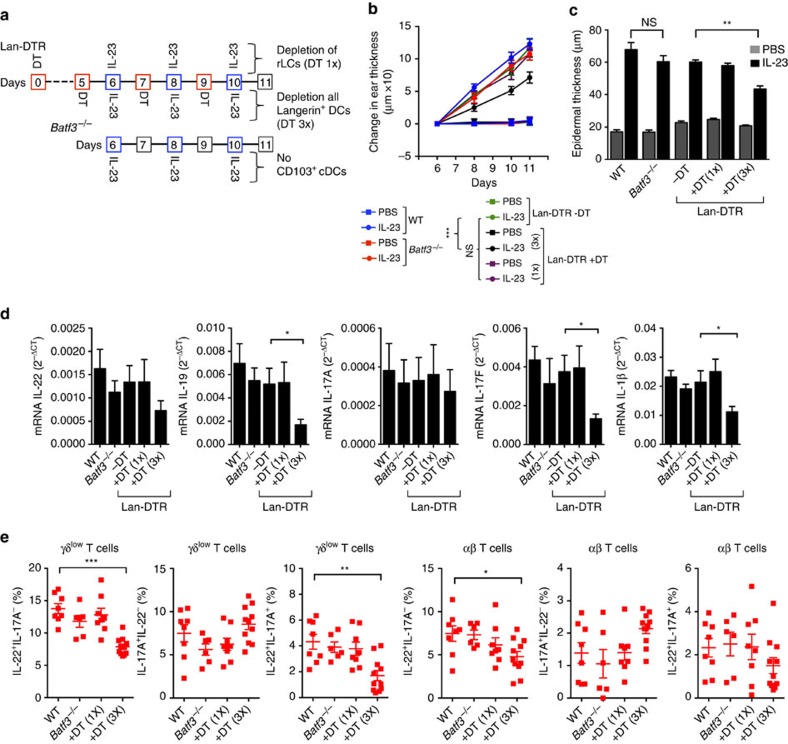
MHC-II^++^ LCs contribute to IL-23-induced inflammation. (**a**) Protocol for DT treatment for depletion of only rLCs or all Langerin-expressing DCs in Langerin-DTR/EGFP (Lan-DTR) mice undergoing ear injections with IL-23 (top). In parallel, the ears of WT and *Batf3*^*−/−*^ mice were also injected with IL-23 (bottom). PBS was injected into the ears of appropriate control mice at the same time as IL-23 (not indicated). (**b**) Changes in the thickness of PBS- and IL-23-injected ears of WT and *Batf3*^*−/−*^ mice and of similarly injected ears of Lan-DTR mice following treatments with DT once or three times or not at all, as diagrammed in **a**. (**c**) Epidermal thickness of ear skin on day 11. (**d**) Expression of mRNAs encoding proteins as indicated versus *Gapdh* mRNA in IL-23-injected ears on day 11. (**e**) Frequencies of T cells expressing IL-22 and/or IL-17A in IL-23-injected ears on day 11. The cells were activated and stained as in Fig. 2e. The data are displayed from one experiment representative of two or three experiments with a minimum of seven mice total per group (**b**; mean±s.d.; pairwise statistical comparisons were done at day 6 using mice from all the experiments); or two experiments with five mice per group of IL-23-injected mice, except for the +DT(1x) group, which had three mice (**c**); or two experiments with a minimum of six mice total per group (**d**); or two or three experiments with a minimum of six mice total per group (**e**). The data are presented as mean±s.e.m., unless otherwise noted. NS, not significant; *P<0.05, **P<0.01, ***P<0.001 (unpaired Student's *t*-test).

To test a possible role for LCs, we took advantage of Langerin-DTR/EGFP (Lan-DTR) mice. In the IL-23-injected skin, we found langerin (CD207/EGFP) expression in LCs and CD103^+^cDCs (Fig. 1b and Supplementary Fig. 3a). In the Lan-DTR mice, although all langerin^+^ DCs are depleted after a single DT treatment, dermal langerin^+^ DCs reappear within 3–4 days, whereas in the absence of inflammation rLC-like cells take 17–20 days to be restored^35^,^36^. Repeated administration of DT maintained depletion of all LCs and langerin^+^ dermal cDCs (Supplementary Fig. 6). In parallel, we also used *Batf3*^*−/−*^ mice, which specifically lack CD103^+^ cDCs^1^, a subset of the langerin^+^ DCs present in the dermis. Deletion of *Batf3* did not have a significant effect on ear swelling or epidermal thickness after IL-23 injection, and the levels of mRNAs for inflammatory cytokines in the ears were not significantly diminished in *Batf3*^*−/−*^ mice (Fig. 4a–d).

Similarly, depletion of rLCs using a single injection of DT before injecting IL-23 had little effect on the mediators and markers of inflammation. In contrast, persistent elimination of all langerin^+^ cells by repeated injections of DT, including not only the langerin^+^ dermal cDCs but also the epidermal rLCs and any newly arrived MHC-II^+^ as well as MHC-II^++^ LCs, had significant effects on swelling, epidermal thickness and the relevant cytokine mRNAs in the IL-23-injected ears (Fig. 4a–d). Numbers of γδ^low^ and αβ T cells capable of making IL-22 after activation *ex vivo* were also significantly reduced (Fig. 4e). Taken together with the results from the *Flt3l*^*−/−*^ mice, which lack all langerin^+^ dermal DCs, these data suggest that among the langerin^+^ DCs, the non-rLC-derived MHC-II^++^ LCs, and possibly non-rLC-derived MHC-II^+^ LCs, which were presumably of monocyte origin^4^,^5^,^27^, were important contributors, and necessary for the full inflammatory response to IL-23. Nonetheless, the effect of depleting all langerin^+^ cells did not recapitulate the protection afforded by eliminating all CD11c-expressing cells. Since, in addition, a lack of all cDCs and pDCs in the *Flt3l*^*−/−*^ mice was of limited consequence, the data suggested that, by a process of elimination, moDDCs were another major population important for the IL-23-induced pathology.

**Figure 5:**
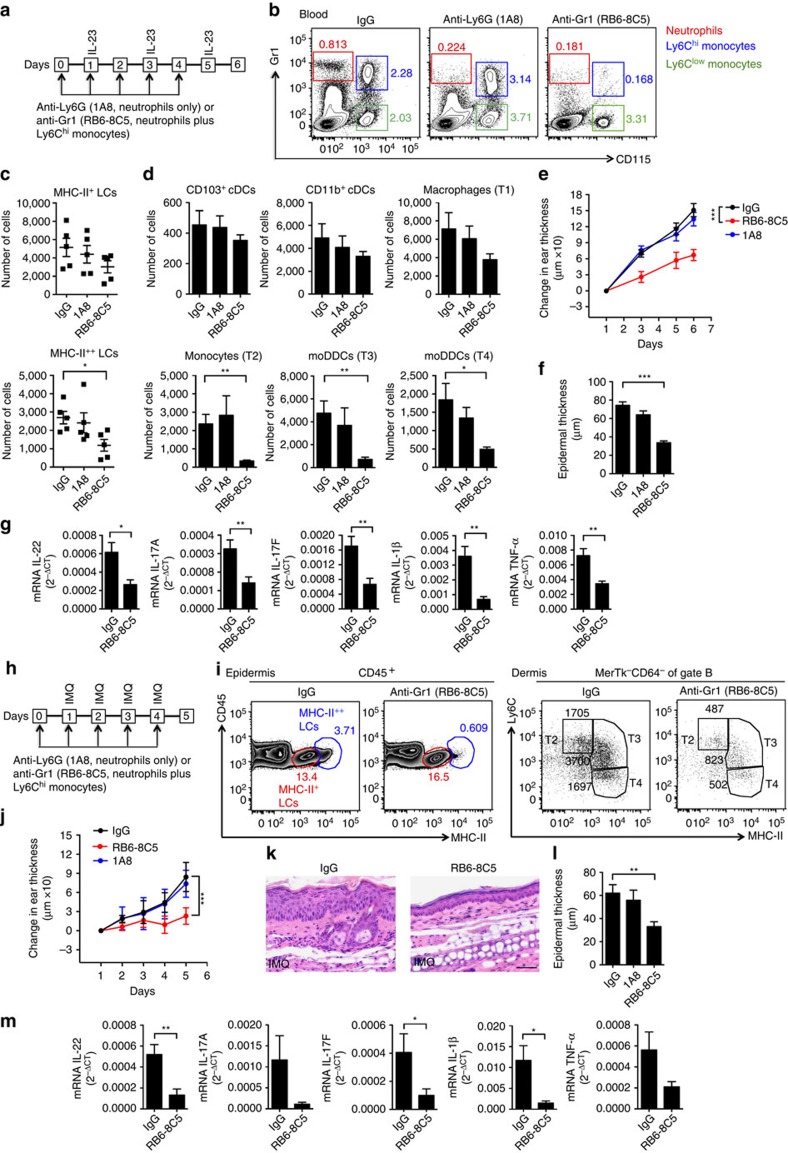
Depletion of Ly6C^hi^ monocytes reduces moDCs and inflammation. (**a**) Protocol for depletion of monocytes plus neutrophils or neutrophils alone in mice whose ears were injected with IL-23. (**b**) Flow cytometry plots of CD45^+^ cells from blood on day 6, following cell depletions per **a**. The numbers indicate percentages of cells within boxes. (**c**,**d**) MHC-II^+^ and MHC-II^++^ LCs and dermal cells per ear on day 6 from IL-23-injected ears. (**e**) Changes in the thickness of IL-23-injected ears. (**f**) Epidermal thickness and (**g**) expression of mRNAs encoding proteins as indicated versus *Gapdh* mRNA on day 6 in IL-23 injected ears. (**h**) Protocol for cell depletions and IMQ application. (**i**) Flow cytometry plots of cells prepared on day 5 from the ears of mice treated as in **h**. The numbers show percentages (epidermis) or absolute numbers (dermis) of cells within the demarcated regions. Gate B is as in Fig. 1e. (**j**) Changes in the thickness of IMQ-treated ears. (**k**) Skin histology on day 5 of IMQ-treated ears. Scale bar, 200 μm. (**l**) Epidermal thickness of ear skin and (**m**) expression of mRNAs encoding proteins as indicated versus *Gapdh* mRNA on day 5 in IMQ-treated ears. The data are from one experiment representative of three with a minimum of four mice total per group (**b**); or two experiments with a total of five mice per group (**c**,**d**); or one experiment representative of two with a total of eight mice per group (**e**; mean±s.d.; pairwise statistical comparison was done at day 6 using mice from all the experiments); or two experiments with a total of five mice per group (**f**); or two experiments with a total of six IL-23-injected and seven IMQ-treated mice per treatment group (**g**,**m**); or one experiment representative of two or three experiments with a minimum of five mice total per group (**i**,**k**); or two experiments with a minimum of seven mice total per group (**j**; mean±s.d.; statistical comparison on day 5); or three experiments with a minimum of five mice total per group (**l**). The data are presented as mean±s.e.m. unless otherwise noted. *P<0.05, **P<0.01, ***P<0.001 (unpaired Student's *t*-test).

To address directly the role of all moDCs, we used antibody-mediated depletion of monocytes as well as *Ccr2*^*−/−*^ mice. Blood monocytes can be identified on the basis of their expression of CSF-1R (CD115), and within the CD115^+^ population, Ly6C defines the classical and non-classical subsets. The available antibodies allowed us to deplete Ly6C^hi^ monocytes plus neutrophils or neutrophils alone (Fig. 5a,b). Depleting neutrophils alone had no effect on the other myeloid subsets or the other components of the inflammatory response in the IL-23-injected ears. The additional depletion of Ly6C^hi^ monocytes led to a partial loss of MHC-II^+^ LCs and dermal macrophages, a major loss of infiltrating monocytes, MHC-II^++^ LCs and moDDCs, and accompanying significant decreases in ear swelling and epidermal thickness, as well as mRNAs for inflammatory cytokines including IL-22, IL-17A, IL-17F, IL-1β and TNF (Fig. 5c–g). The *Ccr2*^*−/−*^ mice, which have profound monocytopenia due to a defect in monocyte egress from the bone marrow, also showed significantly reduced skin pathology and other skin changes (Supplementary Fig. 7a–c). The protective effects of monocyte depletion and *Ccr2* insufficiency were substantially greater than after depletion of LCs using Lan-DTR mice, supporting the importance of both the (monocyte-derived) MHC-II^++^ LCs and moDDCs in the IL-23-induced inflammation.

We also analysed the roles for monocytes/monocyte-derived cells in the model of psoriasis-like inflammation after topical application of IMQ (Fig. 5h–m). As shown in Fig. 5i, IMQ-treated skin contained the same subsets of monocyte-derived cells as we found after injections of IL-23. In addition, monocyte depletion led to decreases in ear and epidermal thicknesses and in mRNAs for the pathogenic cytokines (Fig. 5j–m). Similarly, as shown in Supplementary Fig. 7d–f, the components of the IMQ-induced dermatitis were diminished in the *Ccr2*^*−/−*^ mice. Together, these data support our hypothesis that moDCs are necessary for robust psoriasis-like skin inflammation in these two models.

**Figure 6:**
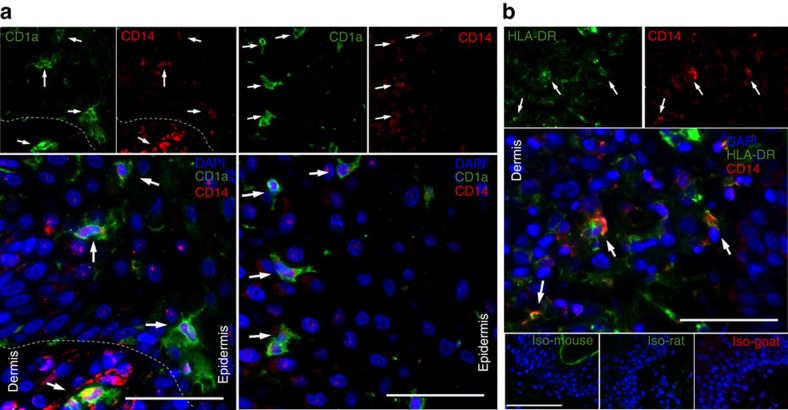
moDCs are found in psoriatic skin. (**a**) Indirect immunostaining of human psoriatic skin for CD1a and CD14. The dermis and epidermis are demarcated by the white dashed lines. Single-colour images are shown above the merged images. The left and right panels are from different subjects. Magnifications are × 100 for the upper panels and × 200 for the lower panels. (**b**) Indirect immunostaining of the dermis of human psoriatic skin for HLA-DR and CD14. Single-colour images are shown above the merged images. Lower panels show staining using isotype controls in place of primary antibodies. Magnifications are × 100 for the upper panels, × 200 for the middle panel and × 50 for the lower panels. Nuclei are stained using DAPI. Staining is visualized using pseudocolours as indicated, arrows indicate both single- and double-positive cells, and scale bars are 50 μm. Sections are from two (**a**), or one subject (**b**), representative of three subjects.

To identify analogous, monocyte-derived LCs and dermal DCs in human psoriatic skin, we used immunofluorescence to detect CD14 together with CD1a or HLA-DR. Both LCs and inflammatory DCs in human skin can be derived from CD14^+^ cells^8^,^37^. Compared with lesional skin, healthy skin contains only small numbers of CD14^+^ cells, and these are confined to the dermis (Supplementary Fig. 8a). We found moLCs (CD1a^+^CD14^(dim)^) in the epidermis and moDDCs (HLA-DR^+^CD14^(dim to bright)^) in the dermis of lesional psoriatic skin (Fig. 6a,b), consistent with a role for these cells in the human disease. In line with their identity as Langerhans cells, CD1a^+^ cells in lesional epidermis were almost all found to co-express CD207/langerin (Supplementary Fig. 8b).

**Figure 7:**
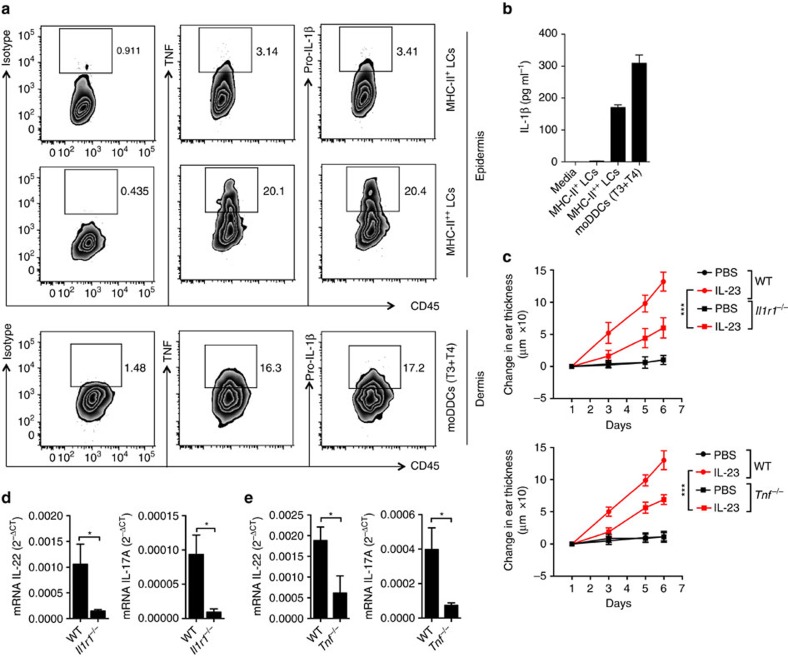
moDCs produce pro-inflammatory cytokines. (**a**) Flow cytometry plots of cells prepared on day 6 from IL-23-injected ears following treatment of the cells *ex vivo* with monensin for 4 h. Surface staining was done as in Fig. 1, followed by intracellular staining for TNF and pro-IL-1β. Numbers indicate percentages of cells within the boxes. (**b**) Measurements of IL-1β in supernatants from cells from IL-23-injected ears on day 6, purified by cell sorting after staining as in Fig. 1 and cultured for 18 h. (**c**) Changes in the ear thickness for WT versus *Il1r1*^*−/−*^ or *Tnf*^*−/−*^ mice injected in the ears with PBS or IL-23 on days 1, 3 and 5. (**d**,**e**) Expression of mRNAs encoding IL-22 and IL-17A versus *Gapdh* mRNA from IL-23-injected ears on day 6. The data are from one experiment representative of two (pro-IL-1β) or three (TNF) experiments with a total of four pro-IL-1β and six TNF mice (**a**); or two experiments with a total of 20 mice (**b**); or one experiment representative of two with a total of eight mice per group (**c**, mean±s.d.; pairwise statistical comparisons were done at day 6 using mice from all the experiments); or two experiments with a total of eight mice per group (**d**); or two experiments with a total of six mice per group (**e**). The data are presented as mean±s.e.m. unless otherwise noted. *P<0.05, ***P<0.001 (unpaired Student's *t*-test).

In considering ways in which moDCs could mediate their pro-inflammatory activities, we analysed cells from the IL-23-injected ears for the production of IL-1β and TNF, cytokines that are important in psoriasis and/or the IL-23 injection model, and are made by DCs in psoriatic skin^9^,^38^,^39^. Both MHC-II^++^ LCs and moDDCs (T3+T4) were producers of IL-1β and TNF in the IL-23-injected skin (Fig. 7a,b). Although we found that small percentages of dermal monocytes and cDCs were also producing these cytokines (Supplementary Fig. 9), the percentages of cytokine-expressing cells together with the subsets' relative abundances indicated that among the cDCs, monocytes and monocyte-derived cells, the MHC-II^++^ LCs and moDDCs (T3+T4) were the principal sources of IL-1β and TNF in epidermis and dermis, respectively.

To assess directly the role of IL-1β and TNF, we injected IL-23 into the skin of *Il1r1*^*−/−*^ and *Tnf*^*−/−*^ mice. Skin thickness, epidermal hyperplasia and mRNA levels of IL-22 and IL-17A were significantly reduced in these mice (Fig. 7c–e). Taken together, these data suggest that moDC-derived IL-1β and TNF are important mediators of IL-23-induced inflammation.

### DCs and/or their precursors require CCR6 for inflammation

**Figure 8:**
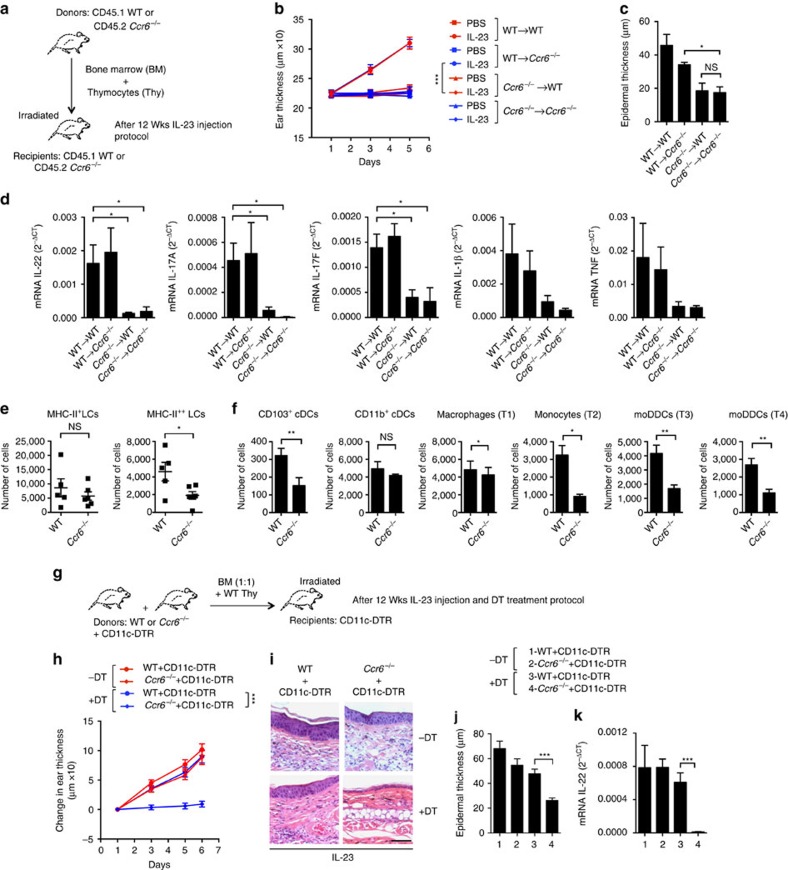
DCs and/or their precursors require CCR6 for IL-23-induced inflammation. (**a**) Protocol for experiments using bone marrow (BM) and thymocyte (Thy) transfers. (**b**) Thickness of PBS- or IL-23-injected ears of transplanted mice following injections on days 1, 3 and 5. (**c**) Epidermal thickness of ear skin, and (**d**) expression of mRNAs encoding proteins as indicated versus *Gapdh* mRNA on day 6 in IL-23-injected ears. (**e**) MHC-II^+^ and MHC-II^++^ LCs per ear on day 6 from IL-23-injected ears. Each symbol represents a sample with cells from two to three ears. (**f**) Dermal cells per ear from IL-23-injected ears. The samples that were analysed contained cells from one or two ears. (**g**) Protocol for experiments using mixed bone marrow chimeras. At 12 weeks, the mice were treated per Fig. 2a. (**h**) Changes in ear thickness in mice transplanted with chimeric bone marrow. (**i**) Skin histology on day 6 of mice as in **h**. Scale bar, 200 μm. (**j**) Epidermal thickness of ear skin, and (**k**) expression of mRNA encoding IL-22 versus *Gapdh* mRNA on day 6 in IL-23-injected ears of mice as indicated by the key. The data are displayed from one experiment representative of two with a total of seven PBS-injected and nine IL-23-injected mice in each transplanted group (**b**; mean±s.d.; pairwise statistical comparison was done at day 6 using mice from all the experiments); or two experiments with a total of three mice per group (**c**); or two experiments with a total of four mice per group (**d**); or four experiments with a total of nine WT and 14 *Ccr6*^*−/−*^ mice (**e**); or three experiments with a total of nine WT and 11 *Ccr6*^*−/−*^ mice (**f**); or one experiment representative of three with a minimum of 10 mice total per group (**h**; mean±s.d.; pairwise statistical comparison was done at day 6 using mice from all the experiments); or one experiment representative of three (**i**); or three experiments with a total of 19 mice (**j**); or two experiments with a total of 18 mice (**k**). The data are presented as mean±s.e.m. unless otherwise noted. NS, not significant; *P<0.05, **P<0.01, ***P<0.001 (unpaired Student's *t*-test).

Given the critical role for both DCs and CCR6 in the IL-23-induced skin inflammation, and the expression of CCR6 on various DC subsets, we investigated a possible role for CCR6 on DCs in this model. Initially, we established that the requirement for CCR6 was within the hematopoietic compartment by transferring WT or *Ccr6*^*−/−*^ bone marrow to lethally irradiated WT and *Ccr6*^*−/−*^ mice (Fig. 8a). Neonatal thymocytes of the matching genotype were transplanted together with bone marrow to reconstitute fully the dermal γ/δ^low^ T cells^40^. At 12 weeks after transplantation, we found that IL-23 injections induced increases in ear swelling, epidermal thickness and cytokine mRNAs in recipients of WT bone marrow, but not in recipients of bone marrow from *Ccr6*^*−/−*^ mice (Fig. 8b–d). Using non-transplanted mice, we found that the *Ccr6*^*−/−*^ mice also showed reduced skin changes in response to IMQ as compared with WT mice (Supplementary Fig. 10).

To determine the activity of CCR6 on DCs, we first analysed the DC subsets in the IL-23-injected ears of *Ccr6*^*−/−*^ mice. We found significant reductions, as compared with WT mice, in the numbers of MHC-II^++^ LCs in the epidermis and numbers of CD103^+^ cDCs, moDDCs, monocytes and macrophages in the dermis (Fig. 8e,f). Although these effects are consistent with a direct role for CCR6 on these cells and/or their precursors, the effects could be the indirect consequences of the lack of inflammation in the *Ccr6*^*−/−*^ mice. Using *Ccr6*^*+/−*^*/*EGFP and *Ccr6*^*−/−*^*/*EGFP knock-in mice, we found expression of *Ccr6* in the MHC-II^++^ LCs, and in some of the CD11b^+^ cDCs but in few of the cells within other DC subsets in the IL-23-injected skin (Supplementary Fig. 11a). We also could not detect significant expression of *Ccr6/*EGFP in CD115^−^CD11c^+^ cells in the blood (Supplementary Fig. 11b).

We investigated a requirement for CCR6 on DCs by transplanting 1:1 mixtures of WT:CD11c-DTR and *Ccr6*^*−/−*^:CD11c-DTR bone marrow into lethally irradiated CD11c-DTR mice (Fig. 8g). In mice transplanted with the *Ccr6*^*−/−*^:CD11c-DTR bone marrow mixture, treatment with DT would be expected to leave CD11c^+^ cells that were all *Ccr6*^*−/−*^, whereas other hematopoietic cells would be both WT and *Ccr6*^*−/−*^. In this way, the functional effects of CCR6 insufficiency would be limited to the populations of CD11c^+^ cells. Mice transplanted with the WT:CD11c-DTR bone marrow mixture served as controls.

Mice that received a mixture of WT and CD11c-DTR bone marrow, either with or without treatment with DT, and mice that received a mixture of *Ccr6*^*−/−*^ and CD11c-DTR bone marrow without being treated with DT showed the typical inflammatory changes after IL-23 injections. However, following the treatment with DT, mice that received the mixture of *Ccr6*^*−/−*^ and CD11c-DTR bone marrow cells were protected from IL-23-induced skin inflammation (Fig. 8h–k). The CD11c-DTR mice were used as recipients for all transplantations so that interpreting the results in DT-treated mice would not be complicated by persistence of radio-resistant CD11c-expressing cells in the irradiated hosts. In addition, DT was injected on days 0, 2 and 4 to maintain depletion of any CD11c-expressing cells. These data suggest that in order to support the IL-23-induced skin inflammation, the DC compartment requires CCR6, either on the DCs themselves or on precursors on which the DCs depend.

CCR6 might have a direct role in recruitment and/or positioning of the MHC-II^++^ LCs and/or their immediate precursor cells, since the MHC-II^++^ LCs express *Ccr6* (Supplementary Fig. 11a). Nonetheless, because depleting the MHC-II^++^ LCs had only a partial effect on preventing the IL-23-induced inflammation, whereas the DT-treated chimeric mice that lacked CCR6 on all CD11c-expressing cells were almost completely protected, we reasoned that CCR6 also had a role in the activity of additional iDCs, that is, the moDDCs, which showed little expression of *Ccr6*/EGFP within the IL-23-injected skin (Supplementary Fig. 11a). We investigated, therefore, a role for CCR6 on monocytes, which serve as the common precursor for MHC-II^++^ LCs and moDDCs.

### Inflammation requires CCR6-dependent monocyte recruitment

**Figure 9:**
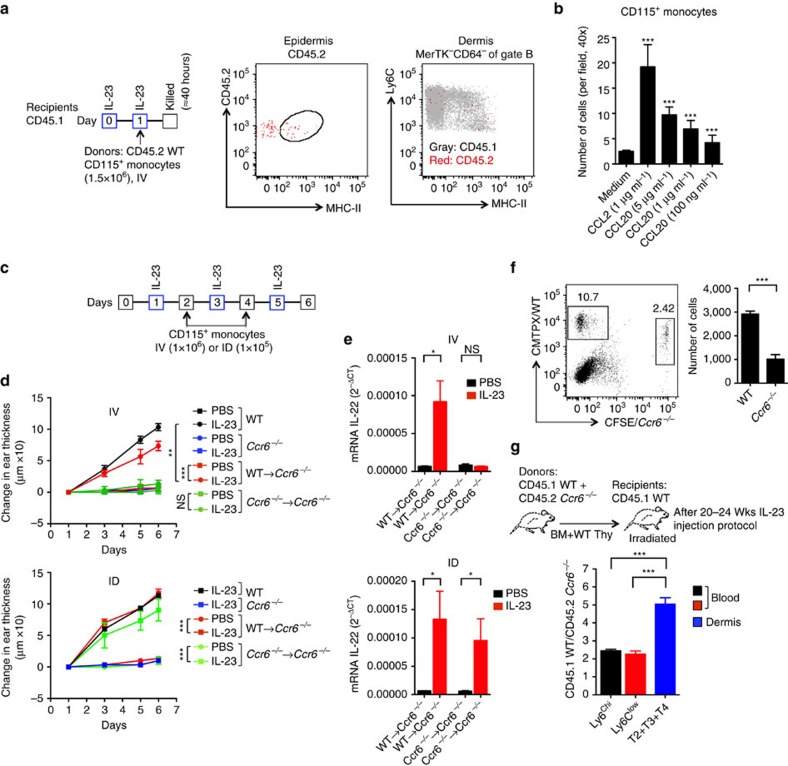
CCR6 is important for monocyte trafficking into inflamed skin. (**a**) Flow cytometry plots of cells prepared from ears 40 h after monocyte transfers as diagrammed on the left, showing CD45.2^+^ cells from epidermis, and an overlay from the dermis analysed as in Fig. 1e, containing cells that express CD45.1 or CD45.2. (**b**) Migration of mouse monocytes to medium alone, CCL2 or CCL20. (**c**) Protocol for monocyte transfers into *Ccr6*^*−/−*^ mice undergoing ear injections with IL-23. (**d**) Ear thickness for WT and *Ccr6*^*−/−*^ control mice, or *Ccr6*^*−/−*^ mice that had received WT or *Ccr6*^*−/−*^ monocytes either intravenously (IV, top) or into the ears (ID, bottom). (**e**) Expression of mRNA encoding IL-22 versus *Gapdh* mRNA in day 6 ears of *Ccr6*^*−/−*^ mice that had received WT or *Ccr6*^*−/−*^ monocytes. (**f**) Flow cytometry plot (left) of cells prepared from IL-23-injected ears from mice injected with monocytes intravenously per **a**. Monocytes were a 1:1 mixture of CMTPX-labelled WT cells and CFSE-labelled *Ccr6*^*−/−*^ cells. Numbers indicate percentages of cells within the boxes. Bar graph shows WT (CMTPX^+^) or *Ccr6*^*−/−*^ (CFSE^+^) monocytes per ear. (**g**) Ratio of WT:*Ccr6*^*−/−*^ blood monocytes and dermal monocytes (T2) and moDDCs (T3+T4) at day 6 in ears injected with IL-23 on day 1, 3 and 5 from mice that had received mixed bone marrow as indicated. The data are from one experiment representative of two with a total of four mice (**a**); or three experiments with a total of six mice (**b**); or one experiment representative of two with a total of six mice per group (**d**; mean±s.d.; pairwise statistical comparisons were done at day 6 using mice from all the experiments); or two experiments with a total of six mice per group (**e**); or one experiment representative of three (**f**, left panel); or three experiments with a total of five mice per group (**f**, right panel); or three experiments with a total of nine and 12 mice used for measurements in blood and dermis, respectively (**g**). The data are presented as mean±s.e.m. unless otherwise noted. NS, not significant; *P<0.05, **P<0.01, ***P<0.001 (unpaired Student's *t*-test).

By injecting CD45.2 monocytes into CD45.1 mice intravenously, we established that blood monocytes were able to give rise to LCs and DDCs in IL-23-injected skin (Fig. 9a). Using the *Ccr6*^*−/−*^/EGFP knock-in mice whose ears had been injected with IL-23, we detected *Ccr6/*EGFP in both Ly6C^hi^ and Ly6C^low^ CD115^+^ blood monocytes (Supplementary Fig. 11c). In addition, we detected mRNA for CCR6 in CD115^+^CD3^−^CD19^−^NK1.1^−^ blood cells from mice whose ears had been injected with IL-23 (Supplementary Fig. 11d), and, parenthetically, we noted that the mRNA was higher in the subset of blood monocytes expressing MHC-II^32^ (Supplementary Fig. 11d). The level of mRNA for CCR6 in the MHC-II^−^ monocytes was approximately 10% of the level in B cells, which express high levels of *Ccr6* (refs 24, 41). Moreover, mouse and human monocytes responded to CCL20 in chemotaxis assays, suggesting expression of functional CCR6 on these cells (Fig. 9b and Supplementary Fig. 11e).

Remarkably, we found that intravenous injection of CD115^+^ blood monocytes from WT mice, but not from *Ccr6*^*−/−*^ mice, into *Ccr6*^*−/−*^ recipients restored the recipients' ability to respond to intradermal injections of IL-23 (Fig. 9d,e), although restoration was not complete. Responses of *Ccr6*^*−/−*^ mice to injections of IL-23 could be re-established by monocytes from either WT or *Ccr6*^*−/−*^ mice, if cells were injected directly into the ears (Fig. 9d,e). We tested the ability of WT versus *Ccr6*^*−/−*^ monocytes to traffic into IL-23-injected ears by co-injecting differentially labelled cells intravenously. Wild-type monocytes accumulated in the skin significantly better than monocytes from *Ccr6*^*−/−*^ mice (Fig. 9f). In addition, we transplanted irradiated CD45.1 WT mice with a 1:1 mixture of bone marrow cells from CD45.1 WT and CD45.2 *Ccr6*^*−/−*^ mice and subsequently injected ears of these mice with IL-23. As shown in Fig. 9g, there was a moderate enrichment of WT versus *Ccr6*^*−/−*^ monocytes, both Ly6C^hi^ and Ly6C^low^, in the blood, and a significant, additional enrichment in WT versus *Ccr6*^*−/−*^ monocytes and moDDCs (T2+T3+T4) in the IL-23-injected ears. Meaningful ratios of CD45.1:CD45.2 cells could not be calculated for LCs in the transplanted mice given that the rLC cells are radioresistant. Taken together, our data suggest that one role for CCR6 in IL-23-induced skin inflammation is the recruitment of blood monocytes that serve as precursors for moDCs.

## Discussion

In the current study, we demonstrated that psoriasis-like inflammation requires moDCs, which produce the critical cytokines, TNF and IL-1β, and depend on CCR6 for their optimal accumulation in the skin. DCs are abundant within psoriatic skin, and pDCs, resident cDCs and iDCs have all been proposed to be important for the initiation and/or maintenance of disease. DCs are thought to act in psoriasis principally by their ability, through cell-to-cell contact and/or secreted products, to activate T cells^7^,^9^. Subsets of iDCs have been described in lesional skin, including cells that produce TNF and iNOS^42^, CD11c^+^CD1c^−^ cells that are able to activate T cells and polarize them to Th1 and Th17 cells *in vitro*^8^, and so-called slanDCs^43^—although some of these cells might be considered ‘inflammatory monocytes'^39^.

Recently, lymphocytes that depend on and/or are activated by IL-23, and that produce IL-17A/F and/or IL-22 have been recognized as critical mediators of psoriasis and other autoimmune diseases^7^,^20^,^44^. For psoriasis, antibodies against the p40 subunit of IL-12/IL-23, the p19 subunit of IL-23, IL-17A and TNF are effective therapies^7^,^15^,^45^. Given the convincing evidence for a direct contribution of IL-23 to psoriasis, we concentrated on a reductionist model that relies on intradermal injections of IL-23 alone, in which the dermatitis depends on the subsequent expression of IL-22 and IL-17A (refs 11, 16, 17, 38). In addition, we used the IMQ model, a commonly used and IL-23-dependent model that was developed following the observation that patients treated with Aldara cream experience flares of psoriasis^18^,^46^,^47^. In the IMQ model, both LCs and dermal DCs have been reported to be sources of the IL-23 that activates IL-17A- and IL-22-producing Th and γδ^low^ T cells^14^,^47^,^48^. Together, these models demonstrate components of the pathology and pathophysiology of the human disease.

In our earlier studies, we found that IL-23-induced skin inflammation depended on CCR6 (ref. 17), the chemokine receptor expressed on all IL-17A/F- and IL-22-producing T cells^28^,^49^. Those data suggested, however, that CCR6 was not important for recruiting Th17 cells into the skin, and although CCR6 likely mediates migration of IL-17A/22-producing γδ^low^ T cells from dermis into epidermis after IL-23 injection, it is not known whether this migration is required for IL-23-induced pathology^31^. In the absence of a definitive explanation for protection in the *Ccr6*^*−/−*^ mice, we chose to investigate roles for DCs—since subsets of DCs are known to express CCR6 (refs 3, 23, 25, 26, 27, 29) and, following IL-23 injections, the accumulation of CD11c^+^ cells in the skin was blunted in the *Ccr6*^*−/−*^ mice^17^.

In the current study, using *Itgax* (CD11c)-DTR mice, we found that although the IL-23 injection model bypasses the requirement for DC-derived IL-23, CD11c-expressing cells were necessary for the skin inflammation, including the edema, acanthosis and production of pathogenic cytokines. Nonetheless, *Flt3l*^*−/−*^ mice lacking most cDCs, and Lan-DTR mice treated with DT in order to eliminate only rLCs showed limited or no protection, respectively, against IL-23-induced inflammation. We concluded, therefore, that neither Flt3L-dependent DCs nor rLCs were necessary for a robust inflammatory response. This left moDCs as the principal candidates for the pro-inflammatory cells within the CD11c-expressing population.

Among the DC populations that increased dramatically in the inflamed skin were MHC-II^++^ LCs and moDDCs—populations that are virtually absent in control mice. Experiments with Lan-DTR mice suggested that the MHC-II^++^ LCs contributed significantly to the IL-23-induced inflammation. Bone marrow transfer experiments showed that the MHC-II^++^ LCs could arise both from radio-resistant precursors, possibly rLCs, as well as from bone marrow-derived precursors. Further, our monocyte depletion and transfer experiments demonstrated that MHC-II^++^ LCs arose principally from newly recruited monocytes, consistent with published data showing that monocyte precursors can give rise to LCs under conditions of LC depletion and/or injury/inflammation^4^,^5^,^27^.

As compared with the inflammation-associated, monocyte-derived LCs that were described previously^4^, the MHC-II^++^ LCs accumulating after IL-23 injections displayed some novel features, including high expression of CD11c and CD86. Notably, the monocyte-derived MHC-II^++^ LCs (and monocyte-derived MHC-II^+^ LCs) that appeared under inflammatory conditions were Ly6C^+^. The MHC-II^++^ LCs also expressed high levels of CD207/langerin and EpCAM. Although this is in contrast to the phenotype described previously for monocyte-derived LCs recruited immediately after ultraviolet irradiation of the skin^4^, others have also reported the early acquisition of CD207/langerin expression in monocyte-derived LCs following inflammatory injury^50^. These data suggest that the inflammatory environment created by injecting IL-23 allows for the rapid expression of the activated LC phenotype by monocyte/blood-borne precursors.

Since removing the MHC-II^++^ LCs was only partially effective in protecting against IL-23-induced inflammation, we hypothesized that other moDCs, namely the moDDCs, must also be important for pathology. We showed that monocytes injected intravenously could give rise to these cells. We directly addressed roles for all the monocyte-derived cells in IL-23-induced and IMQ-induced skin inflammation by depleting Ly6C^hi^ monocytes, which led to large decreases in dermal monocytes and moDDCs and also a significant decrease in MHC-II^++^ LCs—but only a small decrease in MHC-II^+^ LCs. Depletion of Ly6C^hi^ monocytes significantly diminished the inflammatory response, supporting the functional importance of the moDCs. In addition, the experiments with *Ccr2*^*−/−*^ mice were consistent with a role for monocytes/moDCs in driving the IL-23-induced and IMQ-induced skin inflammation. Although it was not possible to deplete moDDCs selectively, that is, without either also affecting other DCs by using CD11c-DTR mice or other monocyte-derived cells by using anti-Gr1 antibody or *Ccr2*^*−/−*^ mice, the effects of monocyte depletion or *Ccr2* insufficiency were substantially greater than after depleting langerin-expressing cells, including the MHC-II^++^ LCs. Taken together, therefore, the data indicate that both populations of moDCs, the moLCs and the moDDCs, contribute to the activities of the CD11c-expressing cells. For IMQ-treated skin, published experiments indicate that infiltrating myeloid cells are not the source of the IL-23 that is required for the IMQ-induced inflammation^13^. Together with our results on the effects of monocyte depletion in the IMQ model, the data indicate a pro-inflammatory role for moDCs other than by producing IL-23, and are consistent with our findings that monocyte-derived cells were important even when IL-23 was supplied by injection. Finally, in support of a role for moDCs in human disease, immunofluorescence of lesional skin from psoriasis patients revealed monocyte-derived LCs and DDCs.

Our results using the *Ccr2*^*−/−*^ mice differ from those published by Bromley *et al*., who showed that the *Ccr2*^*−/−*^ mice developed enhanced inflammation with novel features of atopic dermatitis after intradermal injections of IL-23 (ref. 51). We cannot explain the discrepancies between our findings and those of Bromley *et al*. Undefined environmental differences in the mouse colonies are one possible explanation.

Recent data on the activities of moDCs in immune responses have revealed these cells' abilities to take up antigen and traffic to lymph nodes to activate T cells^32^, and, in particular, to produce inflammatory mediators in tissue^52^,^53^. Just as for cDCs, migration of moDCs to lymph nodes from tissue requires CCR7 (ref. 1). Because experiments using *Ccr7*^*−/−*^ mice indicated no role for the migration of DCs to lymph nodes in our IL-23 injection model, we presumed that the moDCs had their effects locally.

The moDCs can produce TNF and IL-1β (ref. 53), and we found that both moDDCs and the MHC-II^++^ LCs were making these cytokines in IL-23-injected skin, consistent with pro-inflammatory roles for both these populations of moDCs. IL-1β and TNF are important in this model, since both *Il1r1*^*−/−*^ and *Tnf*^*−/−*^ mice showed significantly reduced dermatitis. Previous data have shown that IL-1α and/or IL-1β are important for inducing IL-17A from skin γδ^low^ T cells^20^ and for a component of the inflammation induced by IMQ^22^, and polymorphisms in *IL1B* are associated with psoriasis and psoriatic arthritis^21^. TNF is one of the mediators that supports the production of IL-17A in psoriasis^54^ and is a therapeutic target in this disease.

Our data on the importance and the activities of moDCs in the IL-23 injection model notwithstanding, these are not the cells that initiate the inflammatory response. The moDCs only accumulate after the injection of IL-23, and DCs in the skin do not express the *Il23r* reporter. We showed that among the cells in the ear, expression of *Il23r* was detected only in T cells, and that 18 h after injection of IL-23 we could detect expression of *Il17a* specifically in the γδ^low^ T cells. Collectively, our data suggest that following the injection of IL-23, the γδ^low^ T cells initiate the inflammatory response, with the subsequent accumulation of moDCs. The moDCs release TNF and IL-1β, which in turn amplify the production of IL-17A/F and, importantly, IL-22 from T cells. These latter factors have profound effects on keratinocytes, including activation of STAT3, stimulation of proliferation and mobility leading to acanthosis and dysregulated differentiation, and secretion of antimicrobial peptides and chemokines^16^,^55^.

On investigating how the various populations of moDCs were recruited to the skin, we confirmed our original finding that CCR6 is necessary for inflammation in the IL-23 injection model by using not only *Ccr6*^*−/−*^ mice that we had produced^17^, but also a second strain of *Ccr6*^*−/−*^ mice^41^. Further, we demonstrated that the requirement for CCR6 was limited to the radiosensitive hematopoietic compartment. We had found previously that CCL20 was produced by keratinocytes after the injection of IL-23 in both WT and *Ccr6*^*−/−*^ mice^17^, and we showed here that induction of *Ccl20* did not require CD11c^+^ cells. These data suggest that enhanced expression of the CCR6 ligand is an early event, perhaps in response to the IL-23-mediated production, by γδ^low^ T cells, of IL-17A, which is a known inducer of CCL20 in keratinocytes and other cells^56^. Of additional interest, we have now shown that CCR6 is important not only in the IL-23 injection model, but also in the model using IMQ.

The *Ccr6*^*−/−*^ mice showed diminished numbers of MHC-II^++^ LCs and moDDCs in the IL-23-injected ears. Although this might have simply been the result of an overall lack of inflammation in the *Ccr6*^*−/−*^ mice, this finding was also consistent with a role for CCR6 in the recruitment of these cells and/or a common precursor. Using IL-23-injected *Ccr6*/EGFP-knock-in mice, we were able to detect *Ccr6/*EGFP expression in the MHC-II^++^ LCs and in some of the CD11b^+^ dermal cDCs, but in few if any moDDCs. However, using mice transplanted with a mixture of bone marrows from *Ccr6*^*−/−*^ and CD11c-DTR mice, we established that CCR6 was necessary on DCs and/or their precursors for the IL-23-induced skin inflammation.

Taken together, these data on expression of *Ccr6* and CCL20 suggested that CCR6 might have a direct role in the recruitment of the MHC-II^++^ LCs to the epidermis, which might explain part of the requirement for CCR6 in this model. Consistent with this possibility, convincing studies have shown expression of CCR6 on LCs and/or LC precursors, and suggest that CCR6 is important in the recruitment of human^23^,^29^ and mouse LCs^27^ to epidermis under conditions of injury/inflammation and LC depletion. There are also reports demonstrating roles for CCR6 in the migration and function of other DC populations, including moDCs^26^,^57^. However, the precise role of CCR6 on moDCs has been unclear, and there have been limited data on the expression of CCR6 on precursors and tissue subsets of moDCs.

Because our data suggested that CCR6 was critical not only for the MHC-II^++^ LCs, but also for the moDDCs, in which we failed to detect significant expression of *Ccr6*/EGFP, we investigated CCR6 on blood monocytes that serve as the MHC-II^++^ LCs' and moDDCs' common precursor. Whereas some prior studies have found expression of CCR6 and/or responses to CCL20 on monocytes in mice^58^,^59^,^60^,^61^ or humans^62^, other studies have failed to demonstrate expression and/or activity of CCR6 on these cells^23^,^29^,^41^,^63^. However, *in vivo* experiments have revealed a role for CCR6 in recruitment of monocytes to infected or inflamed lungs^64^,^65^ or inflamed experimental air pouches^58^,^65^. CCR6 was also found to be critical for the accumulation of MHC-II-expressing cells at mucosa and skin, and for blood-borne monocytes to cross-prime CD8^+^ T cells after epithelial immunization^26^, although this study did not directly analyse a role in monocyte trafficking into tissue. A number of studies have also shown diminished accumulation of ‘myeloid' DCs in the inflamed lungs of *Ccr6*^*−/−*^ mice^66^,^67^.

We detected expression of *Ccr6* mRNA and *Ccr6*/EGFP in CD115^+^ blood monocytes from mice whose ears had been injected with IL-23. *Ccr6*/EGFP was detectable in a proportion of both Ly6C^hi^ and Ly6C^low^ cells. It is notable that it was difficult to detect *Ccr6/*EGFP expression in blood monocytes from non-manipulated mice, suggesting that IL-23-induced inflammation may have upregulated monocyte expression of *Ccr6*. We could also detect chemotaxis of purified CD115^+^ mouse blood monocytes or CD14^+^ monocytes from human blood to CCL20. Although both WT and *Ccr6*^*−/−*^ monocytes could restore inflammation if injected directly into the ears of *Ccr6*^*−/−*^ mice, when injected intravenously, only WT, but not *Ccr6*^*−/−*^ monocytes, could restore most of the IL-23-induced inflammation. In addition, experiments using co-injected, differentially labelled monocytes and mice transplanted with a mixture of WT and *Ccr6*^*−/−*^ bone marrow showed a significant advantage for the WT monocytes and/or monocyte-derived cells in accumulating in the IL-23-injected skin. Taken together, our data suggest that one role for CCR6 is in the recruitment of blood monocytes into inflamed skin.

IL-23-induced skin inflammation was virtually eliminated in DT-treated CD11c-DTR mice, *Ccr6*^*−/−*^ mice and mice transplanted with a mixture of *Ccr6*^*−/−*^ and CD11c-DTR bone marrow and treated with DT. Moreover, our findings that injecting WT monocytes intravenously into *Ccr6*^*−/−*^ mice, or injecting *Ccr6*^*−/−*^ monocytes into the ears of *Ccr6*^*−/−*^ mice can restore the inflammatory response to IL-23 would argue that the profound protection observed in *Ccr6*^*−/−*^ mice after IL-23 injections was solely due to deficiencies in activities of monocytes/monocyte-derived cells in the skin. However, although depletion of monocytes and/or MHC-II^++^ LCs had significant effects on the inflammation—much more than did the elimination of Flt3L-dependent cDCs or rLCs—protection against inflammation in monocyte-depleted mice was not absolute, that is, did not fully recapitulate the behaviour of the *Ccr6*^*−/−*^ mice. How might this apparent discordance be explained? One possibility is incomplete elimination of monocytes in the monocyte-depletion experiments, resulting in the persistence of small numbers of active moDCs, such as monocyte-derived MHC-II^++^ LCs. A second possibility is that WT and/or *Ccr6*^*−/−*^ monocytes were injected in sufficient excess in the monocyte transfer experiments so that they obscured (CCR6-dependent) roles for other cells. Our data also do not completely rule out a role for non-monocyte derived DCs, such as a subset of dermal Flt3L-independent CD11b^+^ cDCs, whose activities could not be specifically evaluated experimentally.

In this regard and as noted above, besides expression on the MHC-II^++^ LCs, we found expression of *Ccr6*/EGFP in some dermal CD11b^+^ cDCs. Of possible relevance, a population of colonic CCR6^hi^ CD11b^+^CD103^−^ DCs has been identified that increases Th17 responses and promotes T cell-mediated colitis^68^. Because numbers of CD11b^+^ cDCs do not increase in the IL-23-injected skin, any activity for CCR6 on these cells may be in positioning the cells *in situ*, as has been suggested previously for CCR6 on myeloid cells in skin^28^. In fact, based on patterns of expression of CCL20 and CCR6 in psoriatic skin, it has been proposed that this chemokine/receptor pair is important for DC/T cell clustering^25^. Further, a role for CCR6 on DCs *in situ* might help explain the dramatic protection against inflammation in the *Ccr6*^*−/−*^ mice and in mice whose CD11c^+^ cells are CCR6-deficient in spite of the persistent recruitment of some moDCs into the ears of *Ccr6*^*−/−*^ mice. Other activities of CCR6, such as mediating trafficking of γδ^low^ T cells to the epidermis (see above) may also contribute to inflammation.

In summary, we have shown that psoriasis-like skin inflammation requires moDCs, including MHC-II^++^ LCs and moDDCs. These cells can produce IL-1β and TNF, which we postulate are important components in amplifying inflammation and enhancing the abilities of T cells to make essential downstream pro-inflammatory cytokines. The inflammation requires CCR6-sufficient DCs and/or their precursors, in part due to a role for CCR6 in recruiting blood monocytes. We have diagrammed our view of the steps in the pathogenesis of the skin inflammation in Supplementary Fig. 12. We can speculate that our data may apply to other inflammatory processes driven by similar mechanisms and mediators, helping to inform additional investigations into related human diseases and identify critical cellular and molecular contributors to the pathophysiology that can potentially serve as therapeutic targets.

## Methods

### Human samples

Human normal or psoriatic skin samples were collected at the Department of Dermatology, Medical University of Graz, Austria and analysed under the IRB-approved protocol no. 25-293 ex 12/13 (Ethical Committee of the Medical University of Graz). Elutriated human monocytes were obtained from healthy donors by the Department of Transfusion Medicine, Clinical Center, National Institutes of Health (Bethesda, MD, USA) under an IRB-approved protocol. All human participants signed informed consent.

### Mice

The *Itgax*(CD11c)-DTR/EGFP, Lang-DTR/EGFP (Lan-DTR), *Flt3l*^*−/−*^, *Ccr7*^*−/−*^, IL17A-IRES-GFP-KI*, Il1r1*^*−/−*^ and CD45.1 C57BL/6 mice were purchased from The Jackson Laboratory (Bar Harbor, ME, USA). The *Ccr6*-EGFP^41^ knock-in mice were either from The Jackson Laboratory or from our laboratory^17^. IL-23R-GFP.KI mice were a gift from Vijay K. Kuchroo, Brigham and Women's Hospital, Harvard Medical School, Cambridge, MA, USA^34^, and Langerin-DTR mice were from our laboratory^69^. The *Batf3*^*−/−*^, *Tnf*^*−/−*^ and *Ccr2*^*−/−*^ mice were obtained from Taconic Biosciences (Derwood, MD, USA). The C57BL/6 mice were purchased from the Division of Cancer Therapy, NCI, NIH, or from Taconic Biosciences or from The Jackson Laboratory. The male mice were used in all the experiments. All the mice were housed in sterile cages in a specific pathogen-free facility and used at 8–10 weeks of age. Animal protocols were approved by the Animal Care and Use Committee of the NIAID, NIH.

### IL-23 injection and IMQ treatment protocols

For injections of IL-23, anaesthetized mice had intradermal injections of 500 ng rIL-23 (eBiosciences, San Diego, CA, USA) in 20 μl PBS or 20 μl PBS alone into the dorsal sides of the ears on days 1, 3 and 5 (ref. 17). Depending on the experiment, either one or both ears of a mouse were injected. Ear thickness was measured before the injection on day 1 and thereafter on days 3, 5 and 6, immediately before the next injection or before killing the mice. For applications of IMQ, the ears were treated topically on both sides with a total of 25 mg Aldara cream (3M Health Care, Loughborough, UK) containing 5% IMQ, daily for 4 days. The ear thickness was measured before the application on day 1 and thereafter on days 2, 3, 4 and 5, immediately before the next treatment or before killing the mice. The ear thickness measurements were made at the centre of the ears using a G-1A dial thickness gauge (PEACOCK, Ozaki Mfg., Tokyo, Japan).

### Histological analysis

Sections of formalin-fixed, paraffin-embedded whole ears were prepared and stained with haematoxylin and eosin by HistoServe Inc. (Germantown, MD, USA). The haematoxylin and eosin images were captured using a Zeiss microscope (Jena, Germany) and epidermal hyperplasia was assessed by measuring the thickness of the epidermis from the basal layer to the stratum corneum using iVision software (Biovision Inc., Exton, PA, USA). The epidermal thickness was measured on 10 randomly selected areas per × 2.5 or × 10 field from three to five fields per ear. All the measurements were performed blinded to the treatment groups. The results were first averaged per mouse and then averaged per treatment group for statistical analysis.

### Immunostaining of human skin

Human skin samples were fixed overnight in 4% PFA and embedded in paraffin. For immunofluorescent staining, 4 μm sections were de-paraffinized and heat-induced epitope retrieval was performed in sodium citrate buffer pH 6.0. The sections were blocked with 5% donkey serum (Dako, Carpinteria, CA, USA). For the detection of CD1a, 1:300 dilution of mouse monoclonal antibody, clone O10 (Novus, Littleton, CO, USA) was used followed by a 1:800 dilution of polyclonal donkey anti-mouse Alexa Fluor 488-conjugated antibody (Jackson ImmunoResearch, West Grove, PA, USA). CD207 was detected using a 1:300 dilution of rabbit polyclonal antibody (Sigma-Aldrich, St. Louis, MO, USA) followed by a 1:800 dilution of polyclonal donkey anti-rabbit Cy3-conjugated antibody (Jackson ImmunoResearch). CD14 was detected using a 1:100 dilution of goat polyclonal antibody (Novus) followed by a 1:800 dilution of polyclonal donkey anti-goat Alexa Fluor 647-conjugated antibody (Jackson ImmunoResearch). HLA-DR was detected using a 1:200 dilution of rat monoclonal abtibody, clone YE2/36 HKL (Abcam, Cambridge, UK) followed by a 1:800 dilution of polyclonal donkey anti-rat Alexa Fluor 488-conjugated antibody (Jackson ImmunoResearch). Isotype-matched IgG antibodies against irrelevant antigens were substituted for the primary antibodies to serve as negative controls. Nuclei were visualized using DAPI, and slides were mounted with Fluoroshield (Sigma-Aldrich). Photomicrographs were taken using a Leica DM4000B microscope and processed using LAS V3.8 software (Leica, Wetzlar, Germany).

### Flow cytometry and cell sorting

After harvesting the ears, ventral and dorsal sheets were separated from the cartilage and incubated for 30 min at 37 °C dermal side down in Dispase (Corning Inc., Charlotte, NC, USA) to separate the epidermal and dermal sheets. The epidermal and dermal sheets were next incubated separately for 45 min at 37 °C in RPMI 1640 (Invitrogen, Grand Island, NY, USA) containing 0.01% DNAse (Sigma-Aldrich) and, respectively, 0.25 or 0.17 mg ml^−1^ Liberase (Roche Diagnostics, Chicago, IL, USA). The digested ears were passed through a 1 ml syringe to make single-cell suspensions. The lymph nodes or spleens were mechanically disrupted using a syringe plunger in RPMI 1640. The cells were filtered through 70 μm nylon mesh and washed before activation and/or staining. The antibodies used for flow cytometry with mouse cells are listed in Supplementary Table 1 and were used at 1:00 dilutions according to the suppliers' specifications. AccuCount Fluorecent particles (Spherotech, Lake Forest, IL, USA) were used for counting the cells. The analysis of cells after staining was done using an LSR-II flow cytometer (BD Biosciences, San Jose, CA, USA) and the data were analysed using FlowJo software (Tree Star, Ashland, OR, USA). Each flow cytometry plot shows cells prepared from a single ear unless otherwise noted in the figure legend. Cell sorting was done using a FACS Aria (BD Biosciences).

### Intracellular cytokine staining

Intracellular staining was performed after incubating the cells for 4 h with Leukocyte activating cocktail (BD Biosciences) or monensin alone in DMEM containing 2 mM L-glutamine (Invitrogen). Following surface staining, the cells were fixed and permeabilized according to the manufacturer's instructions using the BD Cytofix/Cytoperm Plus Kit (BD Biosciences). Thereafter, the cells were stained overnight with anti-IL-22 and anti-IL-17A or for 1 h with anti-TNF and anti-pro-IL-1β. The analysis of cells by flow cytometry was done as described above.

### RNA isolation and real-time quantitative PCR with reverse transcription

The extraction of RNA from whole ears was performed using RNeasy Fibrous Tissue Mini Kit (Qiagen, Hilden, Germany) according to the manufacturer's instructions. RNA extraction from cells purified by cell sorting was done using PureLink RNA kit (Life Technologies, Carlsbad, CA, USA). Real-time PCR was performed using SuperScript One Step RT–PCR (PCR with reverse transcription) kit (Invitrogen). Primers and probes (FAM labelled) were purchased from Applied Biosystems/Thermo Fisher Scientific (Foster City, CA, USA) to quantify mRNAs. Genes are listed followed by the Applied Biosystem catalogue numbers in parentheses. *Gapdh (Mm99999915_g1)*, *Il22 (Mm01226722_g1), Il17a (Mm00439619_m1), Il17f (Mm00521423_m1), Il19 (Mm01288324_m1), Tnf (Mm00443258_m1), Il1b (Mm00434228_m1), Csf2 (Mm01290062_m1), Il23a (Mm00518984_m1), Ccl20 (Mm01268754_m1), Ccr6 (Mm99999114_s1), s100a9 (Mm00656925_m1) and s100a7 (Mm01218201_m1)*. The reactions were run on an Applied Biosystems 7900HT system using the standard protocol provided by Invitrogen. The expression of mRNAs was normalized to *Gapdh* mRNA by calculating 2^−ΔCt^. The threshold cycle at the limit of detection for *Il22* mRNA was 35 and for other cytokine mRNAs was 40, and when mRNAs were undetectable by RT–PCR these cycle numbers were used for calculating 2^−ΔCt^.

### Depletion of dendritic cells

For depletion of all dendritic cells, *Itgax*(CD11c)-DTR/EGFP mice were injected at day 0, 2 and 4 with 5 ng of DT per gram body weight. To deplete rLCs alone or all langerin^+^ DCs, Langerin-DTR/EGFP (Lan-DTR) mice were injected with 5 ng of DT per gram body weight once or three times, respectively, as described in the ‘Results' section.

### Depletion of monocytes and neutrophils

To deplete monocytes and neutrophils, the mice were injected intraperitoneally with 500 μg of anti-Gr1 (clone RB6-8C5, BioXCell, West Lebanon, NH, USA) on days 0, 1, 2, 3 and 4 (ref. 13). To deplete neutrophils alone, the mice were injected intraperitoneally with 500 μg anti-Ly6G (clone 1A8, BioXCell) using the same schedule.

### Adoptive transfer of monocytes

Monocytes were purified from mouse spleens or peripheral blood mononuclear cells. Peripheral blood mononuclear cells were prepared using Lympholyte-Mammal Cell Separation Media (Cedarlane, Burlington, NC, USA). For some experiments, CD19^+^ cells were depleted using CD19 Microbeads and the remaining cells were stained for CD115, and, for purposes of excluding cells, for CD3 and NK1.1. For other experiments, the cells were stained for CD115 and, for purposes of excluding cells, for CD3, CD19 and NK1.1. The CD115^+^ cells were purified by cell sorting using a FACS Aria (BD Biosciences). Injections of blood monocytes were done using either 1 × 10^6^ cells intravenously or 1 × 10^5^ cells into the ear by intradermal inoculation as described in the ‘Results' section.

### Monocyte tracking

For tracking WT and *Ccr6*^*−/−*^ monocytes, CD115^+^ monocytes were purified from spleens of WT and *Ccr6*^*−/−*^ mice using FACS as described above and stained with CellTracker Red CMTPX or CellTrace CFSE (both from Life Technologies) as per the manufacturer's instructions. A total 1 × 10^6^ cells of each type were injected intravenously into WT mice.

In some experiments, 1.5 × 10^6^ monocytes were purified from spleens of CD45.2 WT mice and injected intravenously into CD45.1 mice.

### Chemotaxis

The CD115^+^ monocytes were purified from the spleens of WT mice using FACS as described above. For experiments using human monocytes, the monocytes obtained from healthy donors by elutriation were purified by negative selection using microbeads (Miltenyi Biotec, Bergisch Gladbach, Germany) to >95% purity as judged by staining for CD3 (Clone; OKT3, eBiosciences), CD19 (Clone; SJ25-C1, BD Biosciences) and CD14 (Clone; MΦp9, BD Biosciences). The antibodies were used at 1:100 (CD3) or 1:20 (CD14 and CD19) dilutions according to the suppliers' specifications. The monocytes were used at 5 × 10^5^ cells ml^−1^ in RPMI 1640 supplemented with 0.5% BSA and 25 mM HEPES (chemotaxis medium). Twenty-five microlitres of chemotaxis medium only or containing chemokines (all from PeproTech, Rocky Hill, NJ, USA) was added to the lower compartments of a 48-well micro-chemotaxis chamber (Neuro Probe, Cabin John, MD, USA). Each condition was assayed in triplicate wells. After assembling the apparatus with a 5.0 μm pore size polyvinylpyrolidone-free polycarbonate membrane (Neuro Probe), 50 μl of cell suspension was added to each upper compartment and the apparatus was incubated at 37 °C in 5% CO_2_ for 3 h. After disassembling the apparatus and removing the residual cells from the upper surface, the membrane was fixed with methanol and stained with Giemsa staining kit (Sigma, St. Louis, MO, USA). The cells on the bottom of the membrane were counted in five fields per well under × 40 magnification using a BH2 microscope (Olympus, Waltham, MA, USA).

### Transplantation of bone marrow

CD45.2 WT or CD45.2 *Ccr6*^*−/−*^ mice were irradiated with a split dose of 1,300 RAD (two doses of 650 RAD each, 4 h apart). Two hours after irradiation, the mice received 5–8 × 10^6^ neonatal WT or *Ccr6*^*−/−*^ thymocytes intravenously. The next day, the irradiated mice received 6 × 10^6^ bone marrow cells from CD45.1 WT or CD45.2 *Ccr6*^*−/−*^ mice by intravenous injection. In other experiments, 6 × 10^6^ bone marrow cells from CD45.2 WT bone marrow cells were transferred to irradiated CD45.1 WT mice following the same protocol. The transplanted mice were used for experiments after 12 weeks.

### Transplantation of mixed bone marrow

To study the effect of CCR6 deficiency on dendritic cells and moDC accumulation, CD45.2 *Itgax(*CD11c)-DTR/EGFP mice were irradiated with a split dose of 1,300 RAD (two doses of 650 RAD each, 4 h apart). Two hours after irradiation, the mice received 5–8 × 10^6^ WT neonatal thymocytes intravenously. The next day, the irradiated mice received 6 × 10^6^ total bone marrow cells from CD45.2 *Itgax(*CD11c)-DTR/EGFP mice plus either CD45.2 *Ccr6*^*−/−*^ mice or CD45.2 WT mice in 1:1 ratios by intravenous injection. The transplanted mice were used for experiments after 12 weeks. In experiments to evaluate competitive accumulation of *Ccr6*^*−/−*^ versus WT monocyte-derived cells in blood and IL-23-injected ears, the CD45.1 WT mice were irradiated with a split dose of 1,300 RAD (two doses of 650 RAD each, 4 h apart). Two hours after irradiation, the mice received 5–8 × 10^6^ WT neonatal thymocytes intravenously. The next day, the irradiated mice received 6 × 10^6^ total bone marrow cells from CD45.2 *Ccr6*^*−/−*^ plus CD45.1 WT mice in 1:1 ratios by intravenous injection. The transplanted mice were used for experiments after 20–24 weeks.

### IL-1β ELISA

The sorted cells were cultured in DMEM containing 10% FBS, 2 mM L-glutamine and 100 U ml^−1^ penicillin and streptomycin for 18 h. The cell supernatants were collected and IL-1β was measured using mouse IL-1β Ready-SET-Go ELISA kit (eBiosciences) according to the manufacturer's instructions.

### Statistical analysis

The total numbers of mice used in experiments were chosen based on our previous experience evaluating inflammation-associated changes in skin injected with IL-23 (ref. 17), as well as pilot experiments. Although mice used in different experimental groups were matched as closely as possible in order to minimize artifacts, there was no formal randomization. Blinding was done for the analysis of epidermal thickness (see above), but not for other analyses. No exclusion/inclusion criteria were applied for the analyses. Numbers of experiments do not refer to technical replicates. Statistical analysis by two-tailed unpaired Student's *t*-test was conducted using Prism software (GraphPad Software, La Jolla, CA, USA). The results are expressed as mean±s.e.m. or mean±s.d. as indicted in the figure legends. Groups shown and subjected to *t*-tests contained *n*≥5 unless noted otherwise in the figure legend. *P*<0.05 was considered as significant. The *P* values are denoted in figures as follows: *P<0.05, **P<0.01, ***P<0.001 and NS, not significant. No corrections were made for multiple comparisons.

### Data availability

The authors declare that all the data supporting the findings of this study are available within the paper (and its Supplementary Information files) or can be obtained from the authors on reasonable request.

## Additional information

**How to cite this article:** Singh, T. P. *et al*. Monocyte-derived inflammatory Langerhans cells and dermal dendritic cells mediate psoriasis-like inflammation. *Nat. Commun.* **7,** 13581 doi: 10.1038/ncomms13581 (2016).

**Publisher's note:** Springer Nature remains neutral with regard to jurisdictional claims in published maps and institutional affiliations.

## Supplementary Material

Supplementary Information Supplementary Figures 1-12 and Supplementary Table 1
