# Supplementary material for: Monocyte-derived inflammatory Langerhans cells and dermal dendritic cells mediate psoriasis-like inflammation
Source: Nat Commun. 2016 Dec 16;7:13581. doi: 10.1038/ncomms13581 (PMC5171657; doi:10.1038/ncomms13581)
Supplement: Supplementary Information — Supplementary Figures 1-12 and Supplementary Table 1 [file ncomms13581-s1.pdf]

Supplementary Figure 1

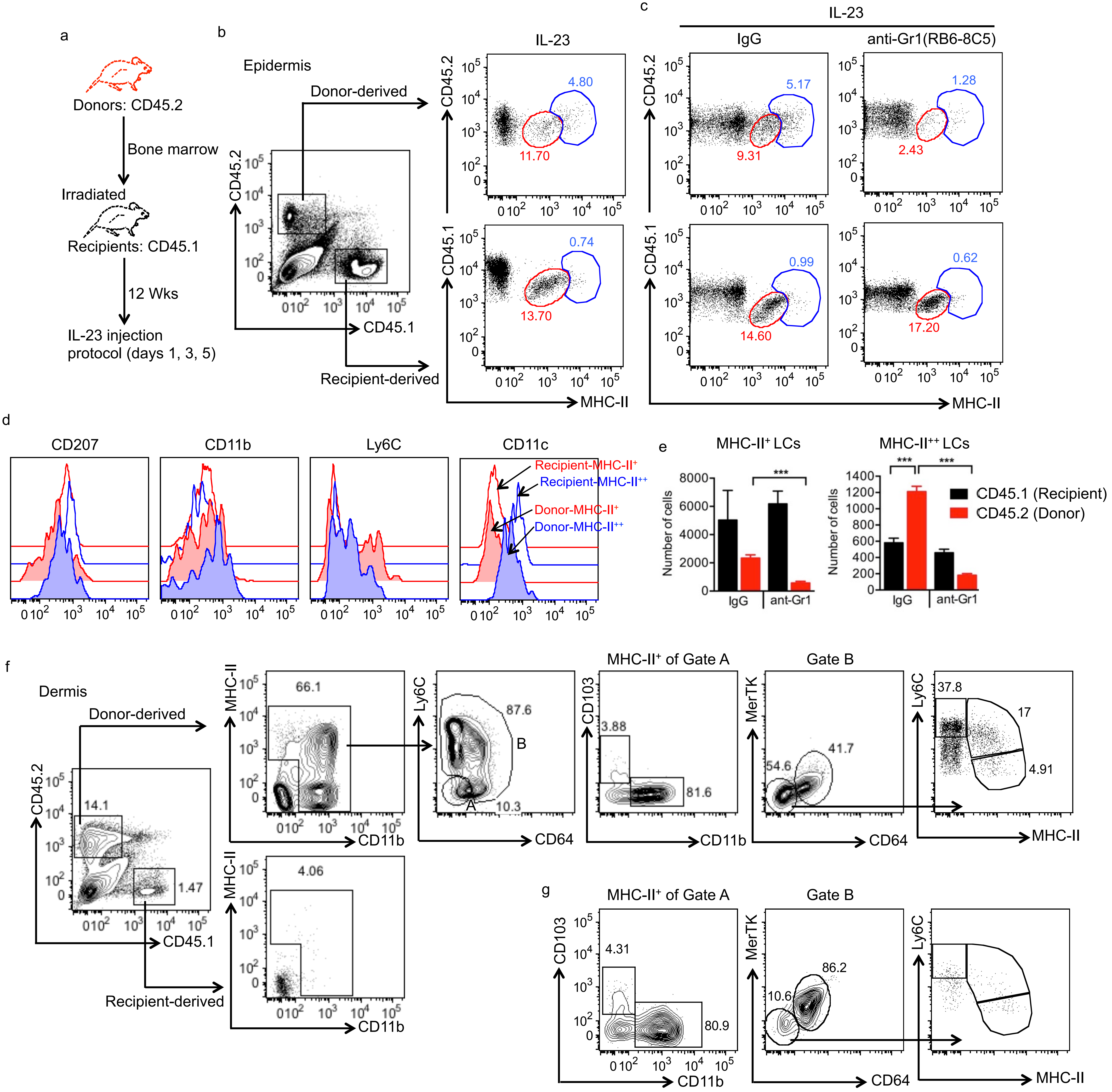

Supplementary Figure 1. Identification of radioresistant and blood derived myeloid cells in epidermis and dermis of IL-23-injected ears. (a) Protocol for transplantation of bone marrow from CD45.2 mice into irradiated CD45.1 mice followed, after 12 weeks, by injections into the ears with PBS or IL-23 on days 1, 3, and 5, and harvesting ears on day 6. **(b)** Flow cytometry plots of cells prepared from epidermis of ears on day 6. Cells from IL-23-injected ears are as labeled. MHC-II^{+} and MHC-II^{++} LCs are identified, respectively, by red and blue demarcated regions. **(c)** Flow cytometry plots of cells prepared on day 6 from epidermis of IL-23-injected ears from mice treated with control IgG or anti-Gr1 (RB6-8C5) antibody. As in b, donor-derived, CD45.2 cells are shown above, and recipient-derived, CD45.1 cells are shown below. **(d)** Flow cytometry histograms showing expression of stained markers as indicated of recipient (CD45.1^{+}, open histograms) and donor-derived (CD45.2^{+}, shaded histograms) MHC-II^{+} (red histograms) and MHC-II^{++} (blue histograms) cells from IL-23-injected ears on day 6. **(e)** Numbers of recipient (CD45.1^{+}) and donor (CD45.2^{+}) MHC-II^{+} and MHC-II^{++} cells prepared on day 6 from epidermis of IL-23-injected ears from mice treated with control IgG or anti-Gr1 (RB6-8C5) antibody. **(f)** Flow cytometry plots of cells prepared from the dermis of IL-23-injected ears on day 6. Numbers indicate percentages of cells in the demarcated regions. **(g)** Flow cytometry plots of donor cells prepared on day 6 from the dermis of IL-23-injected ears from mice treated with anti-Gr1 (RB6-8C5) antibody. Cells are shown from gates A and B as drawn in (f) and Fig. 1e. Numbers indicate percentages of cells in the demarcated regions. Data are from one experiment representative of two or three with a minimum of 4 mice total per group **(b, c)**; one experiment representative of two with a total of 6 mice **(d);** two experiments with a total of 4 IgG and 9 ant-Gr1 mice **(e)**; one experiment representative of three with a total of 6 mice **(f)**; one experiment representative of two with a total of 4 mice **(g).** Data are presented as mean ± SEM. $^{***}P < 0.001$ (unpaired Student's *t*-test).

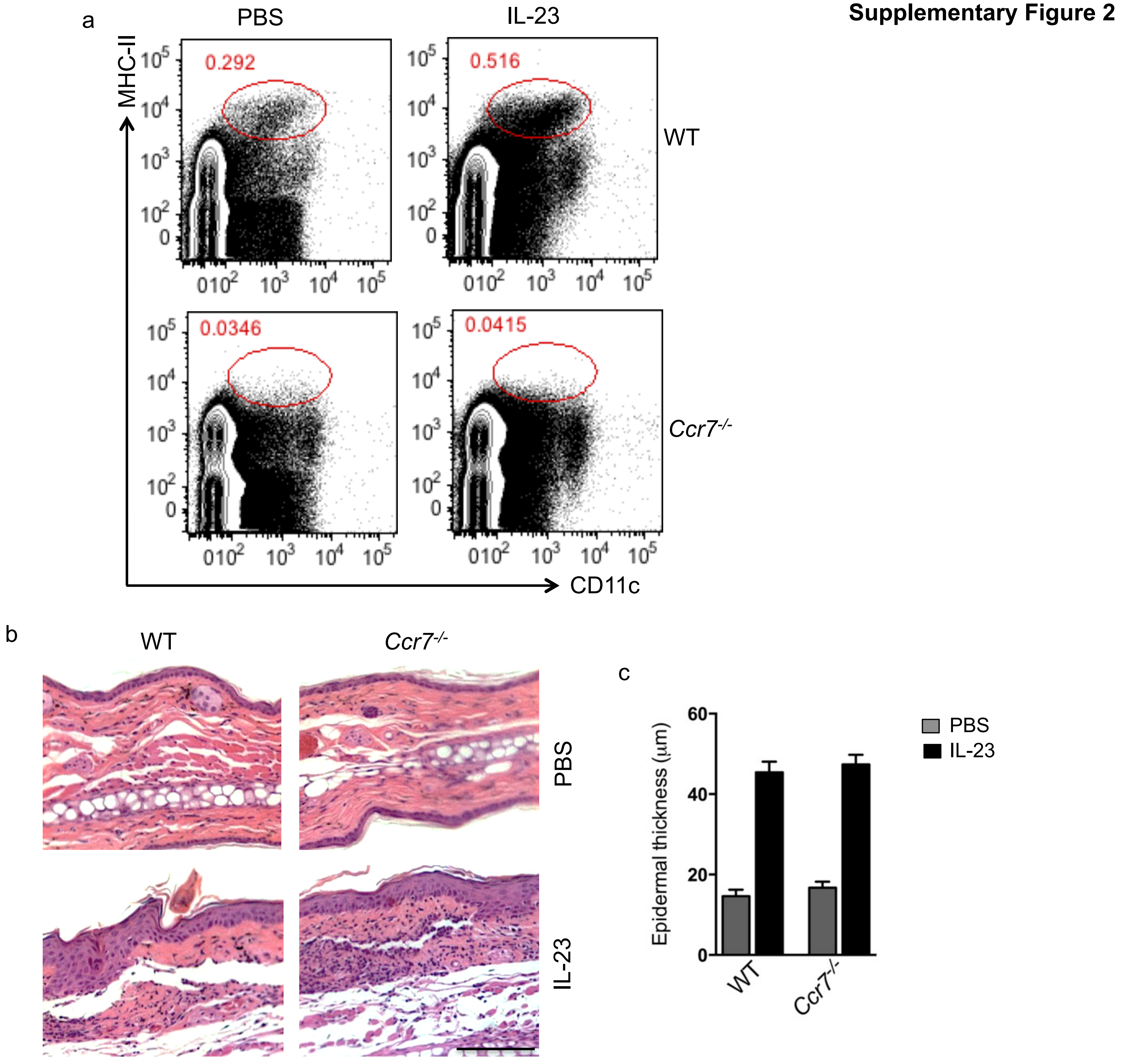

Supplementary Figure 2. Migration of tDCs to skin-draining lymph nodes is not required for IL-23-induced skin inflammation. **(a)** Flow cytometry plots of cells prepared from skin-draining lymph nodes on day 6 after injections of ears of wild-type (WT, top panels) or *Ccr7*$^{-/-}$ (bottom panels) mice with PBS (left) or IL-23 (right) on days 1, 3, and 5. Regions containing tDCs are demarcated in red, and numbers indicate percentages of cells within these regions. **(b)** Skin histology on day 6 of ears of WT (left) and *Ccr7*$^{-/-}$ (right) mice injected with PBS (top) or IL-23 (bottom) on days 1, 3, and 5. Scale bar 200 μm. **(c)** Epidermal thicknesses of ear skin on day 6. Data are from one experiment with skin-draining lymph nodes pooled from 2 mice per group **(a)**; or one experiment representative of two with a total of 2 PBS and 4 or 5 IL-23 mice per group **(b, c).** Data are presented as mean ± SEM. ns; not significant.

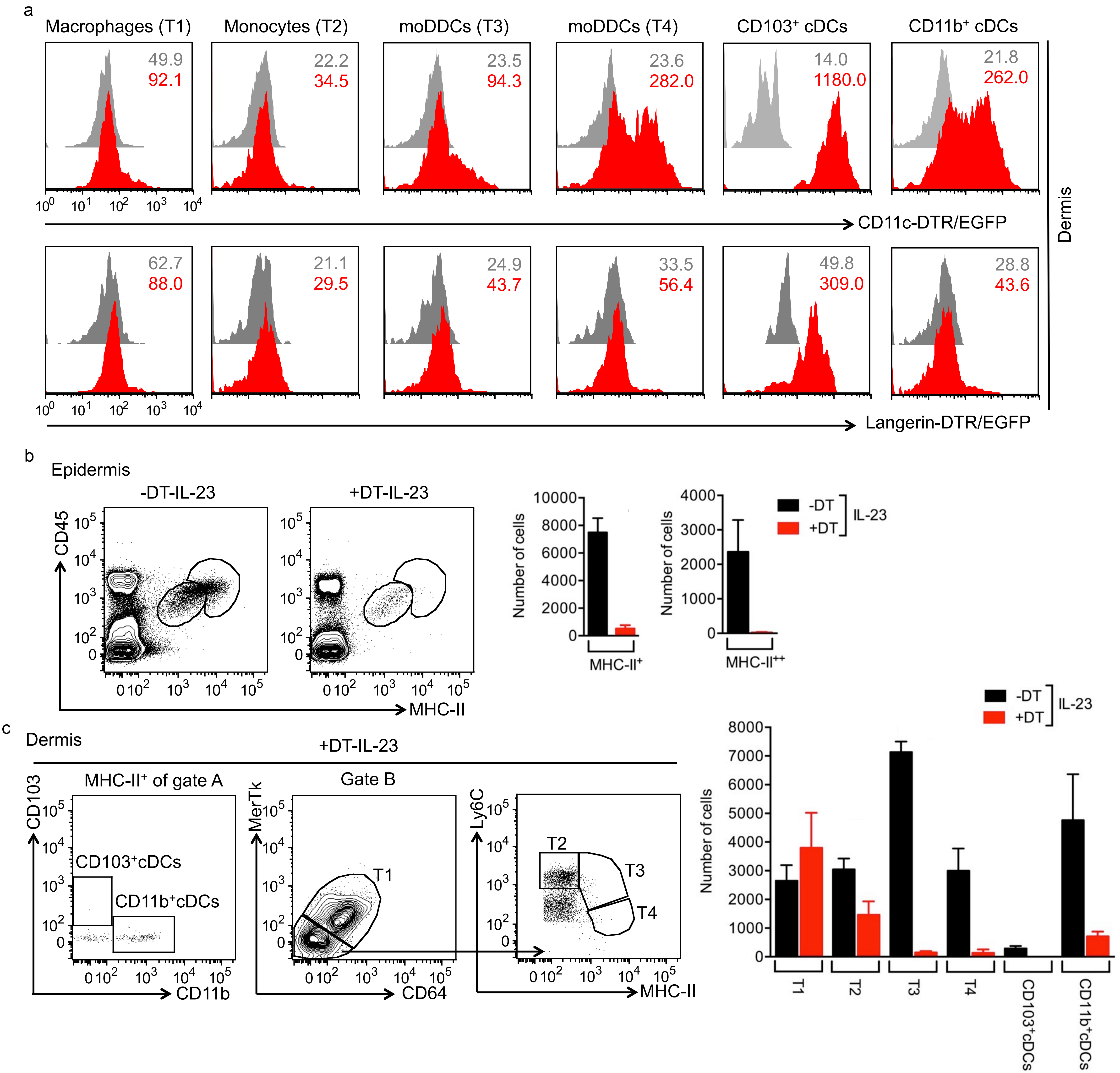
a
Macrophages (T1)
Monocytes (T2)
moDDCs (T3)
moDDCs (T4)
CD103+ cDCs
CD11b+ cDCs
49.9
92.1
22.2
34.5
23.5
94.3
23.6
282.0
14.0
1180.0
21.8
262.0
CD11c-DTR/EGFP
62.7
88.0
21.1
29.5
24.9
43.7
33.5
56.4
49.8
309.0
28.8
43.6
Langerin-DTR/EGFP
Dermis
b
Epidermis
-DT-IL-23
+DT-IL-23
CD45
MHC-II
Number of cells
MHC-II+
MHC-II++
-DT
+DT
IL-23
c
Dermis
+DT-IL-23
MHC-II+ of gate A
CD103
CD103+cDCs
CD11b+cDCs
CD11b
Gate B
MerTk
T1
CD64
Ly6C
T2
T3
T4
MHC-II
Number of cells
T1
T2
T3
T4
CD103+cDCs
CD11b+cDCs

Supplementary Figure 3. Depletion of dermal DCs (cDCs and moDDCs) and epidermal LCs in CD11c-DTR mice after DT treatment. (a) Flow cytometry histograms of cells prepared from the dermis of ears of wild-type (gray) or *Itgax*(CD11c)-DTR/EGFP (red, top panels) or langerin(CD207)-DTR/EGFP (red, bottom panels) mice on day 6 after intradermal injections of IL-23 on days 1, 3, and 5. Numbers show mean fluorescent intensities (MFI). (**b**) Flow cytometry plots (left) or absolute numbers per ear (right) of epidermal cells prepared from IL-23-injected ears on day 6 from CD11c-DTR mice treated with or without diptheria toxin as in Fig. 2a. **(c)** Flow cytometry plots (left) or absolute numbers per ear (right) of dermal cells prepared from IL-23-injected ears on day 6 from CD11c-DTR/WT mice treated with or without diptheria toxin as in Fig. 2a. Cells are shown from gates A and B as drawn as in Fig. 1e. Data are from one experiment representative of two with a minimum of 4 mice total per group **(a)**; or from one representative (flow cytometry plots) or both (bar graphs) of two experiments with a total 2 (-DT) and 5 (+DT) mice **(b, c)**. Data are presented as mean ± SEM. $^{*}P< 0.05$, $^{**}P< 0.01$, $^{***}P< 0.001$ (unpaired Student's *t*-test). ns; not significant.

.

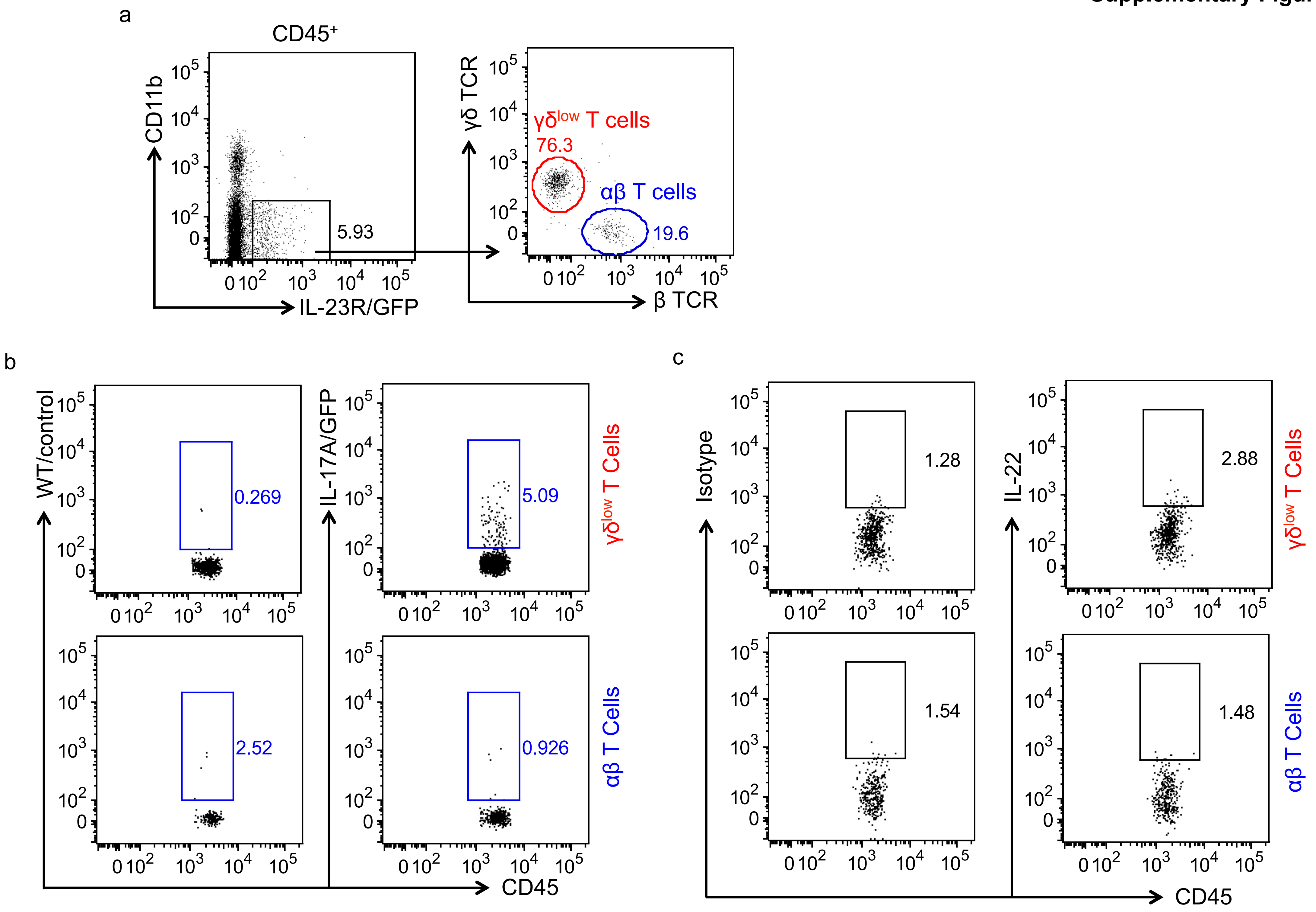

Supplementary Figure 4. $\gamma\delta^{low}$ T cells are the initial responders after IL-23 injection. **(a)** Flow cytometry plots of cells prepared from ears of IL-23R-GFP.KI mice and stained for CD45 and other surface markers as shown. Gating for GFP$^+$ cells was drawn based on the (GFP$^-$) cells from WT mice. **(b)** Flow cytometry plots of dermal cells prepared from ears of WT and IL17A-IRES-GFP-KI mice 18 hours after a single intradermal injection of IL-23. **(c)** Flow cytometry plots of cells prepared from ears of WT mice 18 hours after a single intradermal injection of IL-23, stimulated with leukocyte activation cocktail for three hours and then stained for surface markers and intracellular IL-22. Numbers indicate percentages of cells in the demarcated regions. T cell subsets were identified based on the gating as in Supplementary Fig. 5 (below). Data are from one experiment representative of two with a total 5 mice **(a);** one experiment representative of two with a total 3 mice for detecting cytokines and 2 control mice **(b,c)**.

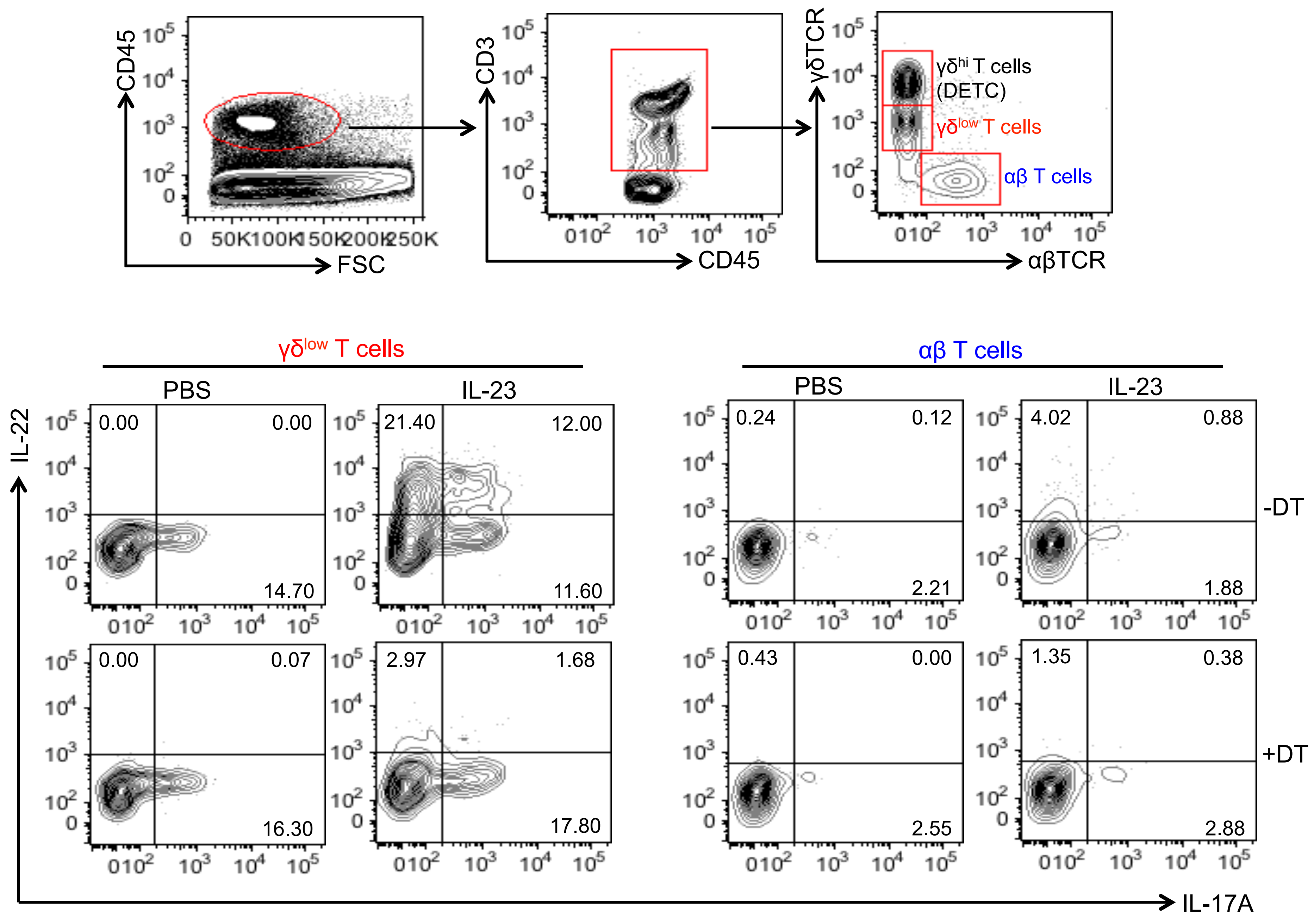

Supplementary Figure 5. Strategy for analyzing IL-22- and IL-17-producing T cells by flow cytometry. Flow cytometry plots of cells prepared on day 6 from PBS- or IL-23-injected ears, stimulated *ex vivo* with leukocyte activating cocktail for four hours, and then stained for surface markers and intracellular IL-22 and IL-17A as shown. Top panels show sequential gating for the identification of γδ^{low} and αβ T cells. Bottom panels show staining for IL-22 and IL-17A in cells from PBS- and IL-23-injected ears of CD11c-DTR mice treated without (-DT) or with (+DT) diptheria toxin. Numbers indicate percentages of cells within the quadrants, and quadrants were drawn based on staining with isotype-matched antibodies. Data are from one experiment representative of many.

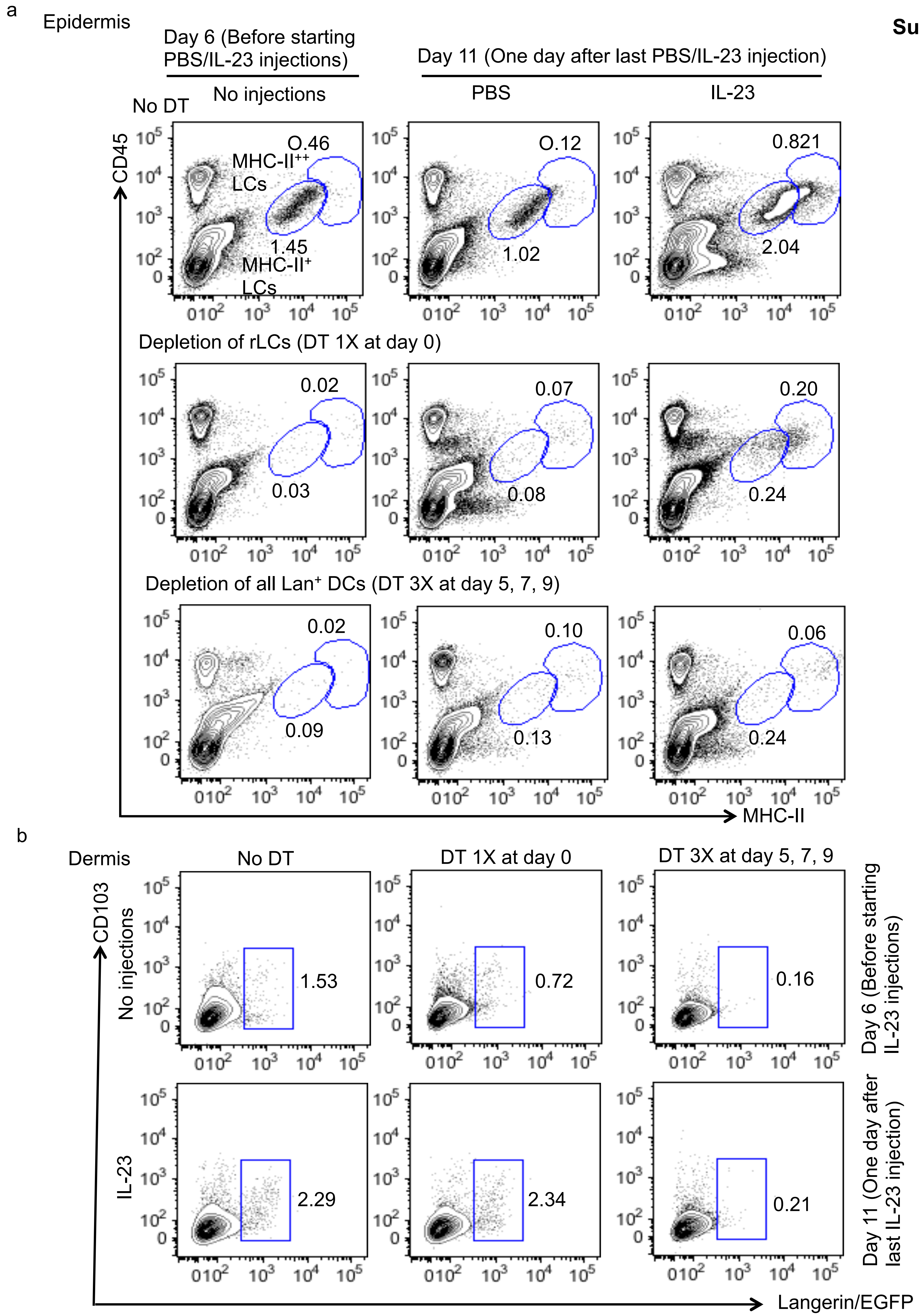
a
Epidermis
Day 6 (Before starting PBS/IL-23 injections)
Day 11 (One day after last PBS/IL-23 injection)
No DT
No injections
PBS
IL-23
CD45
MHC-II++ LCs
MHC-II+ LCs
O.46
1.45
O.12
1.02
0.821
2.04
Depletion of rLCs (DT 1X at day 0)
0.02
0.03
0.07
0.08
0.20
0.24
Depletion of all Lan+ DCs (DT 3X at day 5, 7, 9)
0.02
0.09
0.10
0.13
0.06
0.24
MHC-II
b
Dermis
No DT
DT 1X at day 0
DT 3X at day 5, 7, 9
CD103
No injections
1.53
0.72
0.16
Day 6 (Before starting IL-23 injections)
IL-23
2.29
2.34
0.21
Day 11 (One day after last IL-23 injection)
Langerin/EGFP

Supplementary Figure 6. Subset-specific depletion of langerin-expressing DCs. (a) Flow cytometry plots of cells prepared from the epidermis of ears from Lan-DTR/EGFP mice treated once or three times with diptheria toxin (DT), before or after three injections of PBS or IL-23 according to the protocol in Figure 4a, stained for CD45 and MHC-II. Regions demarcated using blue lines identify MHC-II^{+} and MHC-II^{++} LC subsets, and numbers indicate percentages of cells within these regions. **(b)** Flow cytometry plots of cells prepared from the dermis of ears of Lan-DTR/EGFP mice treated once or three times with DT, before or after three injections of IL-23 according to the protocol in Figure 4a, and stained for CD45, MHC-II, CD11b, and CD103. Only CD45^{+}, MHC-II^{+} and/or CD11b^{+} cells are displayed (see gating in Fig. 1e) . The blue boxes contain langerin/EGFP^{+} cells, and numbers indicate percentages of these cells. Data are from one experiment representative of one or two with a minimum of 4 mice total per group **(a,b)**.

Supplementary Figure 7

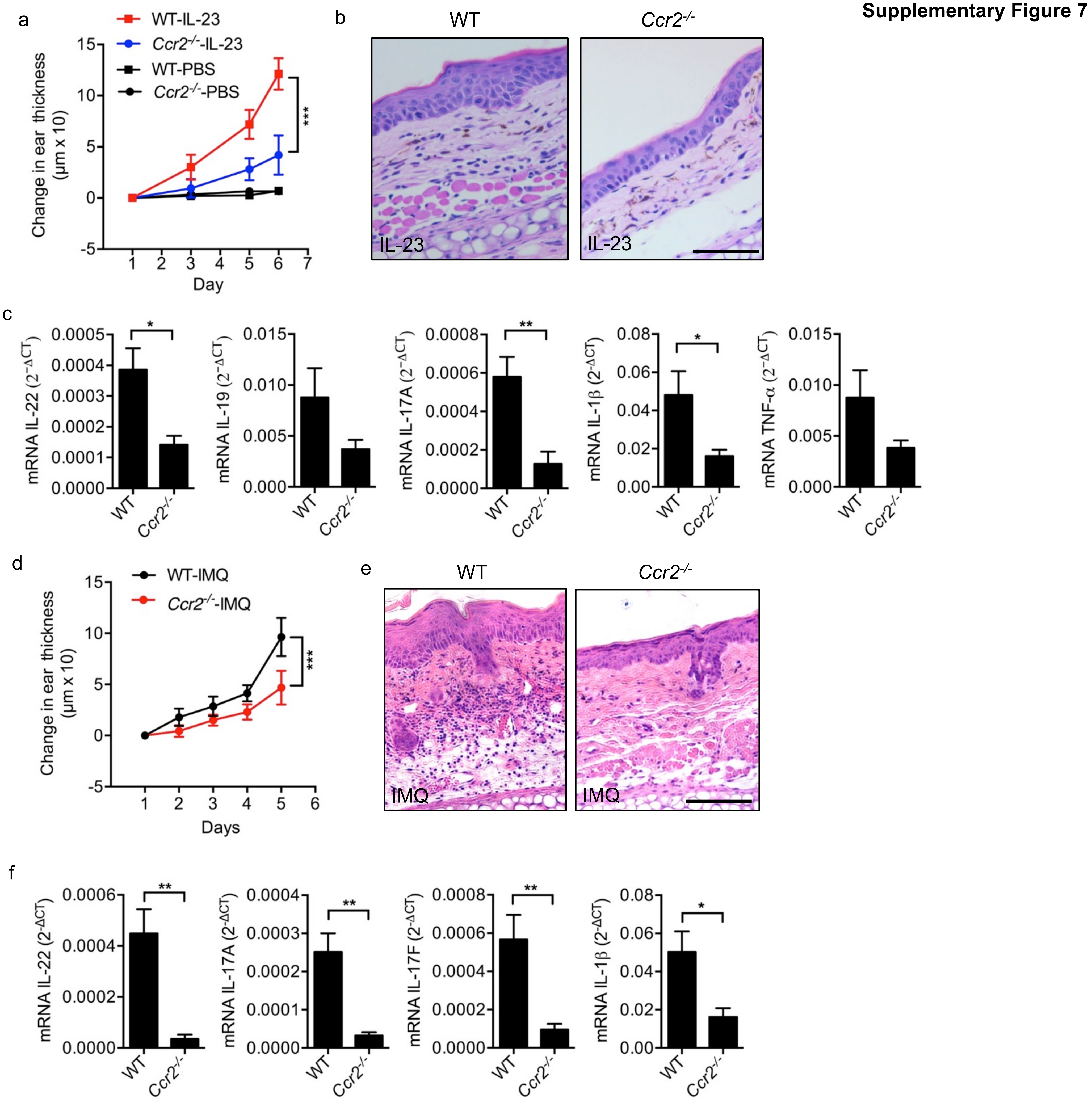

Supplementary Figure 7. IL-23- and imiquimod (IMQ)-induced skin changes are CCR2-dependent. (a) Ear thickness measured for WT and *Ccr2*$^{-/-}$ mice injected in the ears with PBS or IL-23 on days 1, 3, and 5. **(b)** Skin histology on day 6 of IL-23 injected ears. Scale bar 200 µm. **(c)** Expression of mRNAs encoding proteins as indicated versus expression of *Gapdh* on day 6 in IL-23 injected ears. **(d)** Ear thickness measured for WT and *Ccr2*$^{-/-}$ mice treated with IMQ on days 1, 2, 3, and 4. **(e)** Skin histology on day 5 of IMQ-treated ears. Scale bar 200 µm. **(f)** Expression of mRNAs encoding proteins as indicated versus expression of *Gapdh* on day 5 in IMQ-treated ears. Data are from two experiments with a total of 8 mice per group (**a**; mean ± SD; statistical comparison at day 6); or one experiment representative of two **(b)**; or two experiments with total 4 mice per group **(c)**; or two experiments with a total of 7 WT and 8 *Ccr2*$^{-/-}$ mice per group (**d**; mean ± SD; statistical comparison at day 5**)**; or one experiment representative of two **(e)**; or two experiments with a total 6 mice per group **(f)**. Data are presented as mean ± SEM unless otherwise noted. $^{*}P < 0.05$, $^{**}P < 0.01$, $^{***}P < 0.001$ (unpaired Student's *t*-test).

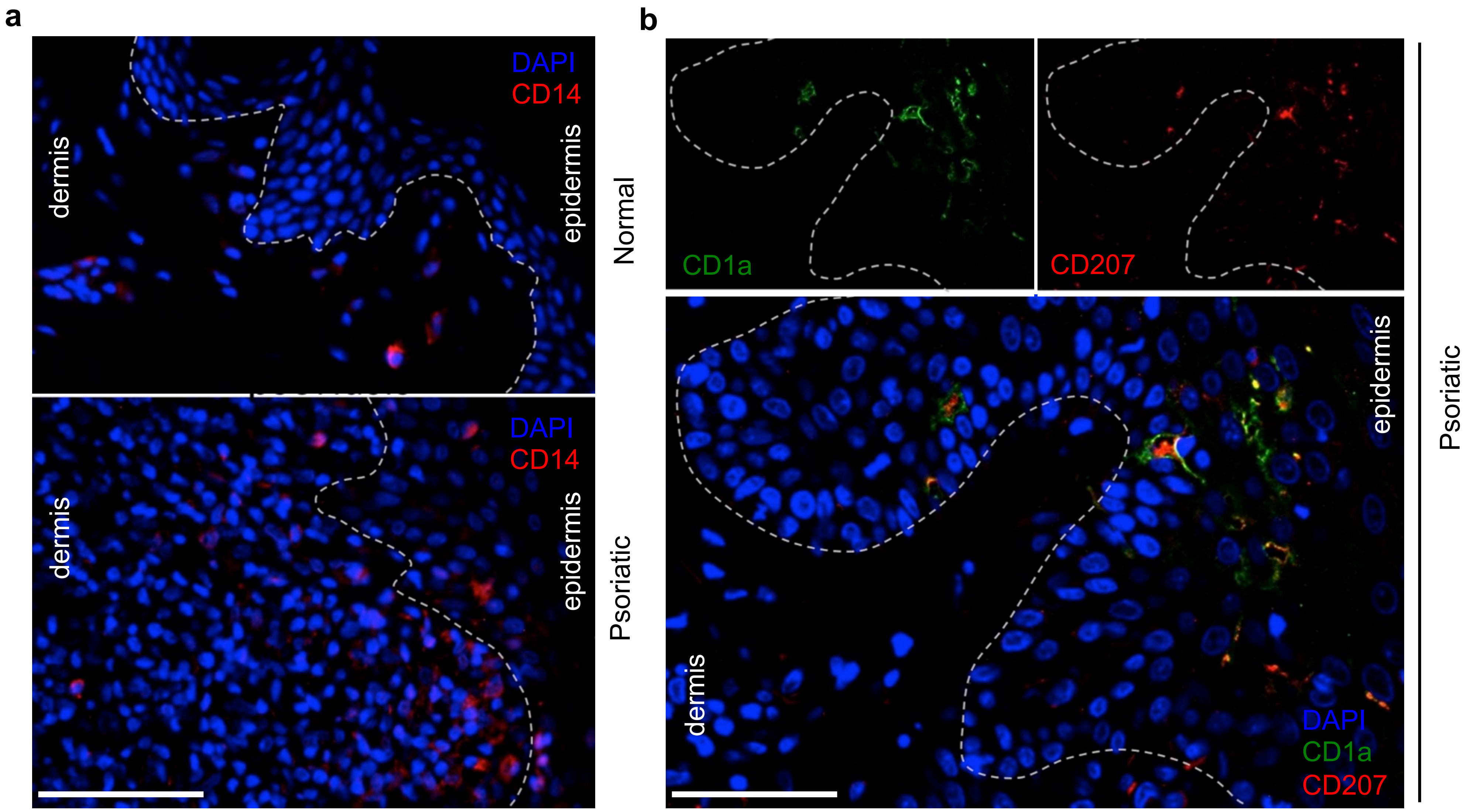

Supplementary Figure 8. Identification of $CD14^+$ cells and LCs in psoriatic skin. (a) Indirect immunostaining of human normal and psoriatic skin for CD14. Dermis and epidermis are demarcated by the white dashed lines. **(b)** Indirect immunostaining of human psoriatic skin for CD1a and CD207. Dermis and epidermis are demarcated by the white dashed lines. Single color images are shown above the merged images. Magnifications are 100x for upper panels and 200x for lower panels. For all panels in a and b nuclei are stained using DAPI. Staining is visualized using pseudocolors as indicated, and scale bars are 50 µm. Each section is representative of one subject (**a, b**).

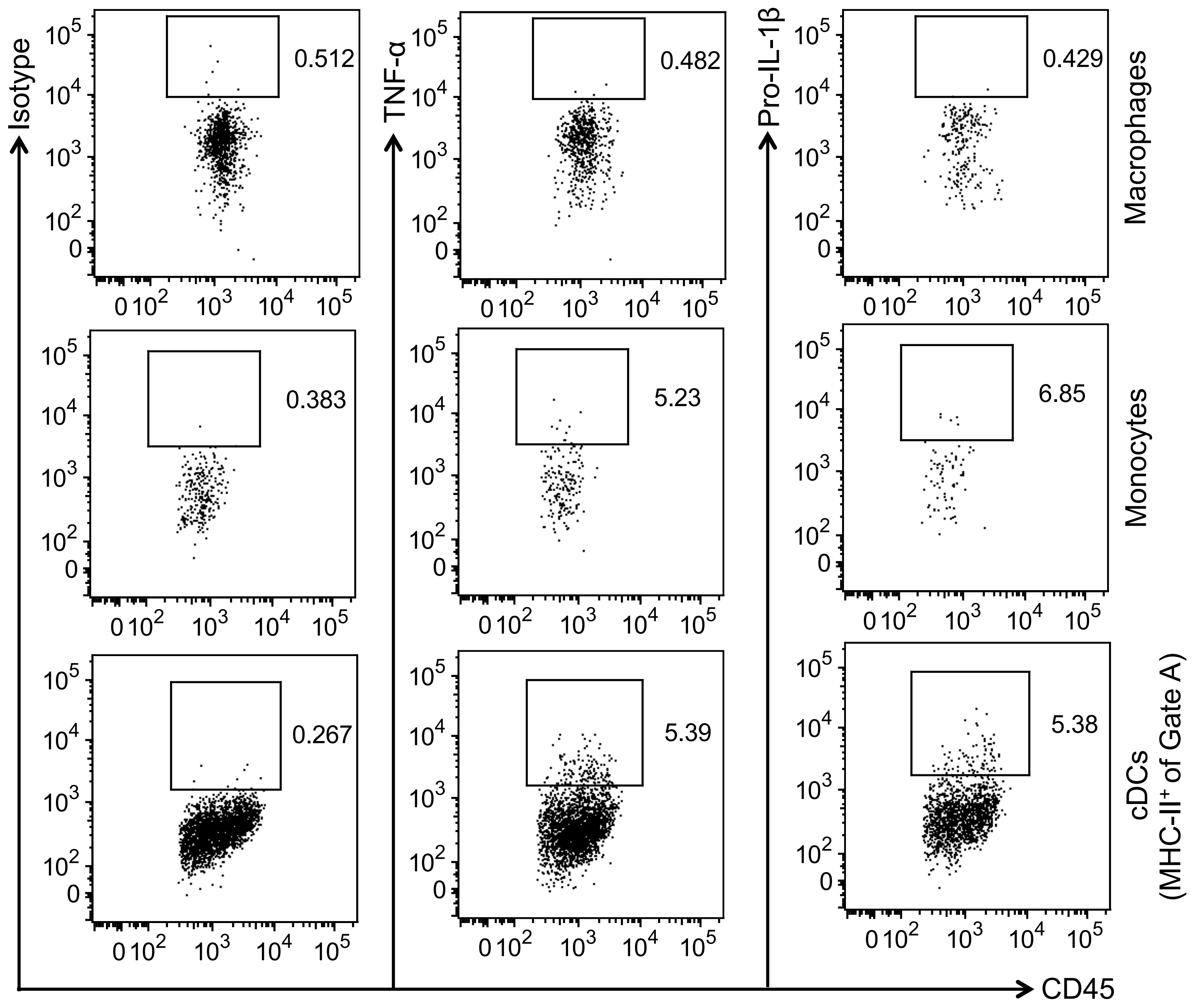

Supplementary Figure 9. Few macrophages, monocytes or cDCs produce TNF-α and IL-1β in IL-23-injected ears. Flow cytometry plots of cells prepared on day 6 from IL-23 injected ears following treatment of the cells *ex vivo* with monensin for four hours. Surface staining was done as in Fig. 1, followed by intracellular staining for TNF-α and pro-IL-1β. Numbers indicate percentages of cells within the boxes. Data are from the same experiment shown in Fig. 7 and are representative of two (pro-IL-1β) or three (TNF) experiments with a total of 4 (pro-IL-1β) and 6 (TNF) mice**.**

Supplementary Figure 10

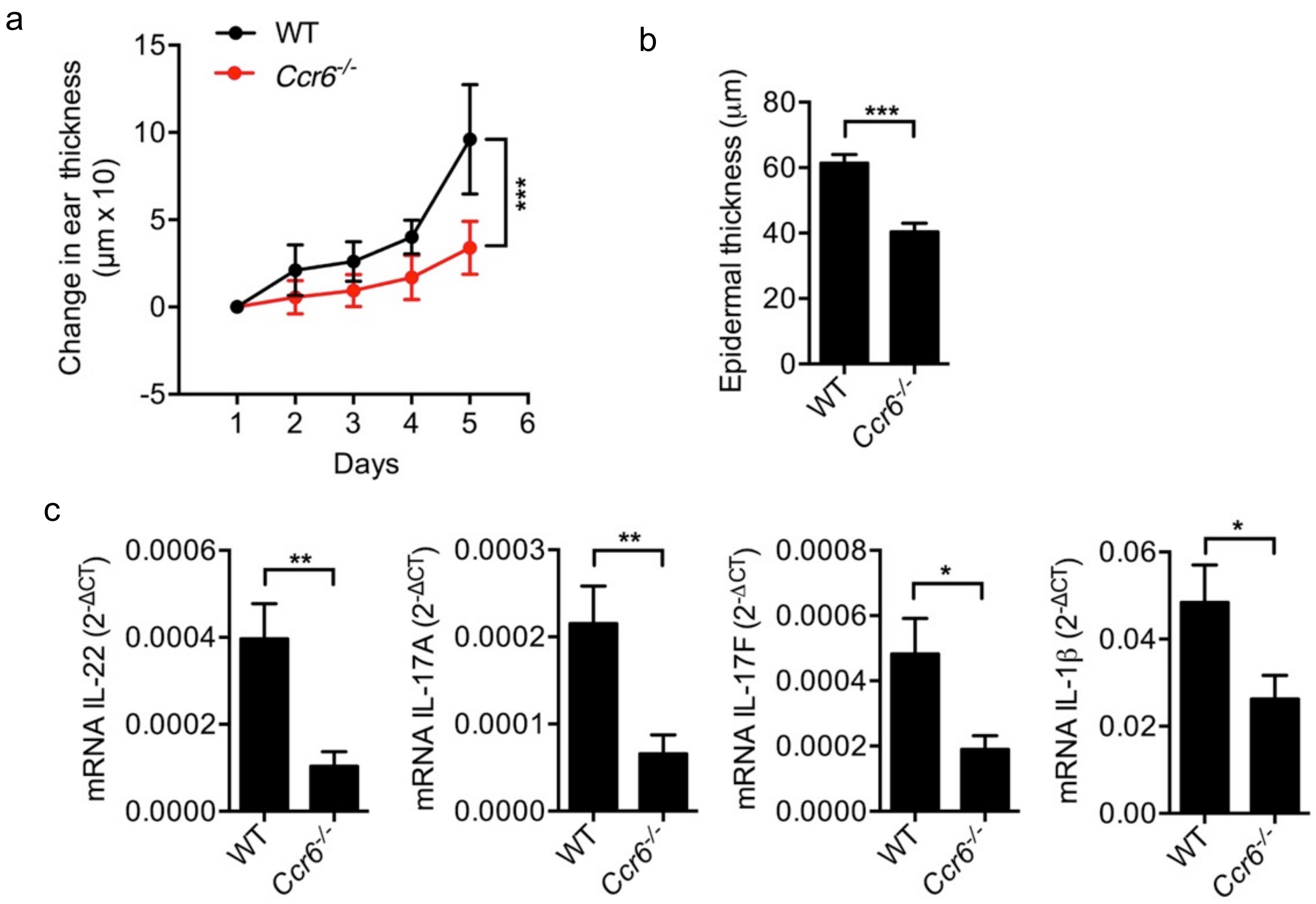

Supplementary Figure 10. IMQ-induced skin changes are CCR6-dependent. (a) Skin thicknesse for WT and *Ccr6*$^{-/-}$ mice treated with IMQ on days 1, 2, 3, and 4. **(b)** Epidermal thicknesse of ear skin on day 5 of IMQ-treated ears of WT and *Ccr6*$^{-/-}$ mice. **(c)** Expression of mRNAs encoding proteins as indicated versus expression of *Gapdh* on day 5 in IMQ-treated ears from WT and *Ccr6*$^{-/-}$ mice. Data are from two experiments with a total of 9 WT and 10 *Ccr6*$^{-/-}$ mice (**a**; mean ± SD; statistical comparison at day 5); or two experiments with a total 8 WT and 6 *Ccr6*$^{-/-}$ mice **(b)**; or two experiments with a minimum of 6 mice total per group **(c).** Data are presented as mean ± SEM unless otherwise noted. $^{*}P < 0.05$, $^{**}P < 0.01$, $^{***}P < 0.001$ (unpaired Student's *t*-test).

Supplementary Figure 11

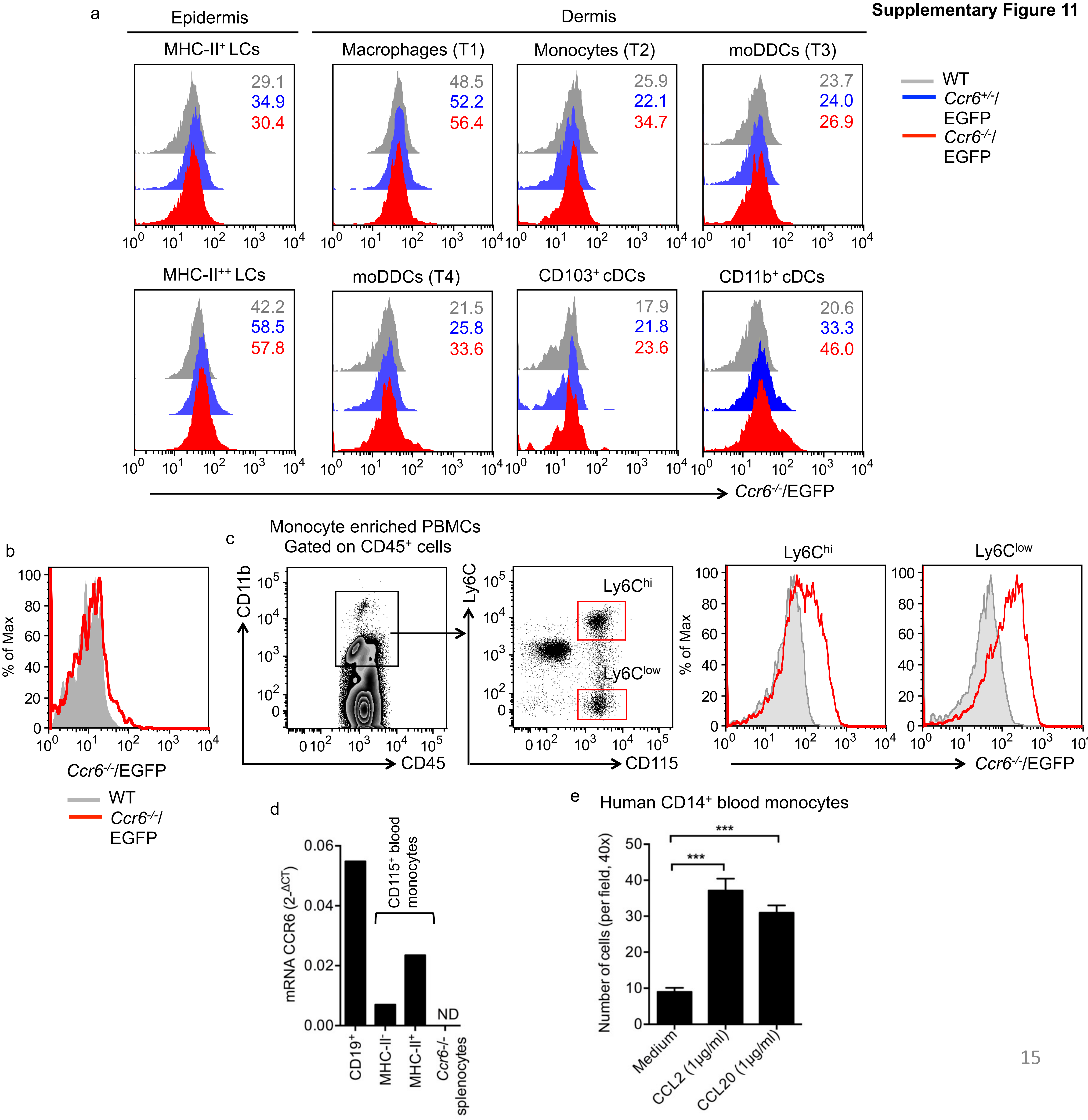

Supplementary Figure 11. ***Ccr6* is expressed on subsets of epidermal and dermal DCs and blood monocytes. (a)** Flow cytometry histograms showing EGFP expression in cells prepared from epidermis and dermis of ears from wild-type (WT, gray), *Ccr6*$^{+/-}$/EGFP (blue) and *Ccr6*$^{-/-}$/EGFP (red) mice on day 6 after intradermal injections of IL-23 on days 1, 3, and 5. Cells were separated for analysis into myeloid subsets according to the gating in Fig. 1. Numbers show mean fluorescent intensities (MFI). **(b)** Histograms showing *Ccr6*$^{-/-}$/EGFP expression on CD115^{-}CD11c^{+} cells from blood of WT (gray) and *Ccr6*$^{-/-}$/EGFP (red) mice. **(c)** Left panels show flow cytometry plots and gating strategy for the identification of Ly6C^{hi} and Ly6C^{low} blood monocytes from WT mice that had been treated as in a, using staining for CD45, CD11b, Ly6C and CD115. Right panels show expression of *Ccr6*$^{-/-}$/EGFP in these cells by using *Ccr6*$^{-/-}$/EGFP mice. Fluorescence of cells from WT (EGFP^{-}) mice is shown in gray as negative controls. **(d)** Expression of mRNA encoding CCR6 in B cells and MHC-II^{-} and MHC-II^{+} monocytes from blood of WT mice that had been treated as in a. mRNA from splenocytes of *Ccr6*$^{-/-}$ mice was used as a negative control. **(e)** Numbers of human monocytes migrating to the far side of a filter in response to medium alone, or 1 μg/ml CCL2 or CCL20 in the lower wells of a micro-chemotaxis chamber. Data are from one experiment representative of two with a total of 6 WT and *Ccr6*$^{-/-}$/EGFP mice and from one experiment with a total of 2 *Ccr6*$^{+/-}$/EGFP mice **(a)**; or one experiment representative of three with a total of 4 mice **(b)**; or one experiment representative of five with a total of 6 mice **(c)**; or one experiment representative of four that demonstrated *Ccr6* mRNA in monocytes, with a total of 60 mice **(d)**; or two experiments using cells from 2 donors **(e)**. ND; not detected

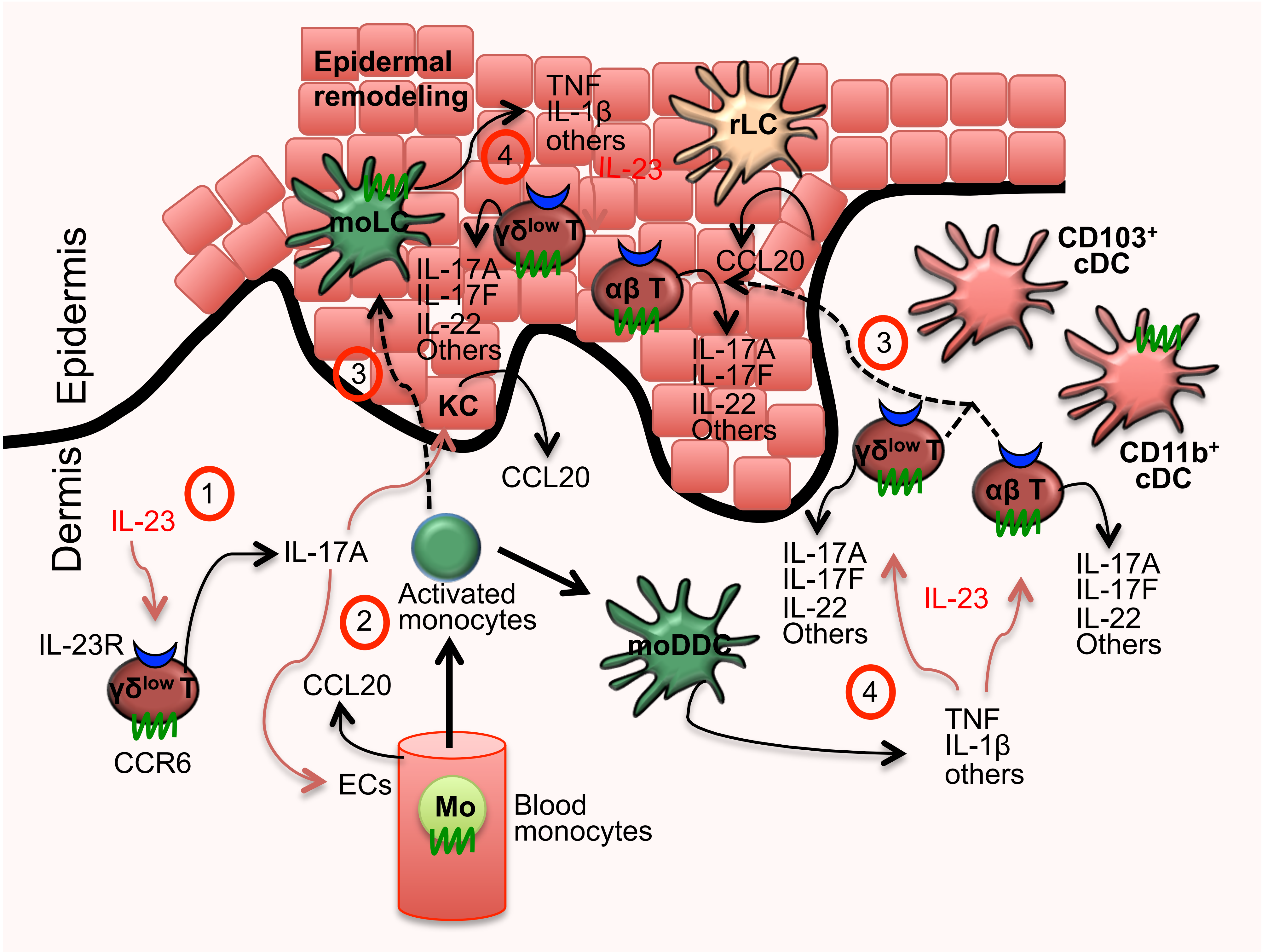

Supplementary Figure 12. moDCs and CCR6 in IL-23-mediated skin inflammation. **1)** We speculate that in response to IL-23, $\gamma\delta^{low}$ T cells produce IL-17A that among other effects induces CCL20 in keratinocytes (KCs) and endothelial cells (ECs). **2)** Activated blood monocytes (Mo) are recruited using CCR6 and give rise to moDCs (moDDCs and moLCs). **3)** moLC precursors and T cells are recruited to the epidermis, also through CCL20/CCR6. **4)** moDCs produce IL-1β and TNF, which together with IL-23 further activate γδ and αβ T cells to produce high levels of IL-17A, IL-17F, IL-22 and other cytokines, as well as chemokines, that recruit additional leukocytes to dermis and epidermis. These factors exacerbate psoriasis-like pathology through their effects on keratinocytes. CCR6 may also contribute to inflammation through roles in positioning DCs and T cells within the dermis and/or epidermis. (rLC, resident LC)

Supplementary Table 1

Antibodies (anti-)	Clone	Fluorochrome	Company	Experiment
CD45	30-F11	eF450	eBiosciences	Cytokine analysis, DC analysis and sorting
CD3	17A2, 145-2C11	AF700, PE	BD Pharmingen	Cytokine analysis, monocyte sorting
γδTCR	GL3	FITC, APC	eBiosciences BD Pharmingen	Cytokine analysis
βTCR	H57-597	PE-Cy5	BD Pharmingen	Cytokine analysis
IL-22	Poly5164	PE	BioLegend	Cytokine analysis
IL-17	TC11-18H10	APC-Cy7	BD Pharmingen	Cytokine analysis
MHC-II	M5/114.152	AF700	eBiosciences	DC analysis and sorting, monocyte analysis
CD11b	M1/70	APC-Cy7	BD Pharmingen	DC analysis and sorting, monocyte analysis
CD11c	HL3, N418	PE-Cy7, PE, FITC	eBiosciences, BD Pharmingen	DC analysis
Ly6C	AL-21, HK1.4	FITC, PE-Cy7, PercpCy5.5	eBiosciences BD Pharmingen	DC analysis and sorting, monocyte analysis
CD64	290322	Percp	R&D Systems	DC analysis and sorting
MerTK	108928	PE	R&D Systems	DC analysis and sorting
CD103	2E7, M290	APC, BV421	eBiosciences	DC analysis and sorting
F4/80		FITC	BD Pharmingen	DC analysis
CD24	M1/69	PE-Cy7	eBiosciences	DC analysis
Epcam	G8.8	APC	eBiosciences	DC analysis
CD8α	53-6.7	PE-Cy7	BD Pharmingen	Cytokine analysis, DC analysis
CD207	eBioL31	PE	eBiosciences	DC analysis
CD115	AF598	APC, PE-Cy7	eBiosciences	Monocyte sorting and analysis
CD86	GL1	PE-Cy7	BD Pharmingen	DC analysis
CD19	1D3	PE	BD Pharmingen	Monocyte sorting
NK1.1	PK136	PE	BD Pharmingen	Monocyte sorting
CD45.1	A20	eF450, FITC	eBiosciences	Chimera analysis
CD45.2	A20, 104	PE-Cy7, FITC, eF450	eBiosciences BD Pharmingen	Chimera analysis
Gr1	RB6-8C5, 1A8	PE-Cy7, FITC	eBiosciences	Monocyte and neutrophil analysis
TNF-α	MP6-XT22	PE-Cy7	eBiosciences	Cytokine analysis
Pro-IL-1β	NJTEN3	PE-Cy7	eBiosciences	Cytokine analysis
